# *In situ* iodine-grafted SPAN *via* a decoupled additive for durable and high-rate Na–S batteries

**DOI:** 10.1039/d6sc06580e

**Published:** 2026-09-18

**Authors:** Mingyue Yang, Xue Li, Qingyi Feng, Yeqing Yang, Xiaocong Shen, Kaizhi Chen, Long Yao, Kunjie Zhu, Xiang-Long Huang, Zhe Hu, Hua-Kun Liu, Yun-Xiao Wang

**Affiliations:** a Institute of Energy Materials Science, University of Shanghai for Science and Technology Shanghai 290000 P. R. China xlhuang_uestc@163.com yunxiaowang@usst.edu.cn; b Guangdong Provincial Key Laboratory of New Energy Materials Service Safety, College of Materials Science and Engineering, Shenzhen University Shenzhen 518060 P. R. China huzhe@szu.edu.cn; c ShenSi Lab, Shenzhen Institute for Advanced Study, University of Electronic Science and Technology of China Shenzhen 518110 P. R. China; d Australian Institute of Innovative Materials, Innovation Campus, University of Wollongong NSW 2500 Australia

## Abstract

Localized high-concentration electrolytes (LHCEs) alleviate polysulfide shuttling and sodium dendrite growth in room-temperature sodium–sulfur (RT Na–S) batteries, yet they remain incapable of resolving the high nucleation barrier for sodium and sluggish sulfur conversion kinetics. Herein, we introduce antimony triiodide (SbI_3_) as a decoupled additive into an LHCE for RT Na–S batteries. The Sb ions promote the formation of a sodiophilic Na_3_Sb interphase, effectively lowering the nucleation overpotential and enabling uniform Na deposition on the anode. Meanwhile, the iodine species are *in situ* grafted onto sulfurized polyacrylonitrile (SPAN) skeletons through their attack on the C≡N bonds, which perturbs the adjacent C–S bonds and thereby weakens the S–S bonds within the covalent –C–S_*x*_–C– chains. The iodine-grafted SPAN cathode is shown to achieve fast redox reaction kinetics and high reversibility. As a result, RT Na-SPAN batteries employing the SbI_3_-modified LHCE deliver a low capacity decay rate of 0.09% per cycle over 2500 cycles at 1.0C as well as a superb rate capability of 685 mA h g^−1^ at 4.0C. This work establishes a new paradigm of *in situ* covalent grafting *via* a bifunctional additive and offers a new approach for the simultaneous engineering of cathode redox kinetics and anode interfacial stability in high-performance metal–sulfur batteries.

## Abstract 2

Localized high-concentration electrolytes alleviate polysulfide shuttling and sodium dendrite in Na–S batteries, yet they remain incapable of resolving the high nucleation barrier for sodium and sluggish sulfur conversion kinetics.

## Graphical abstract

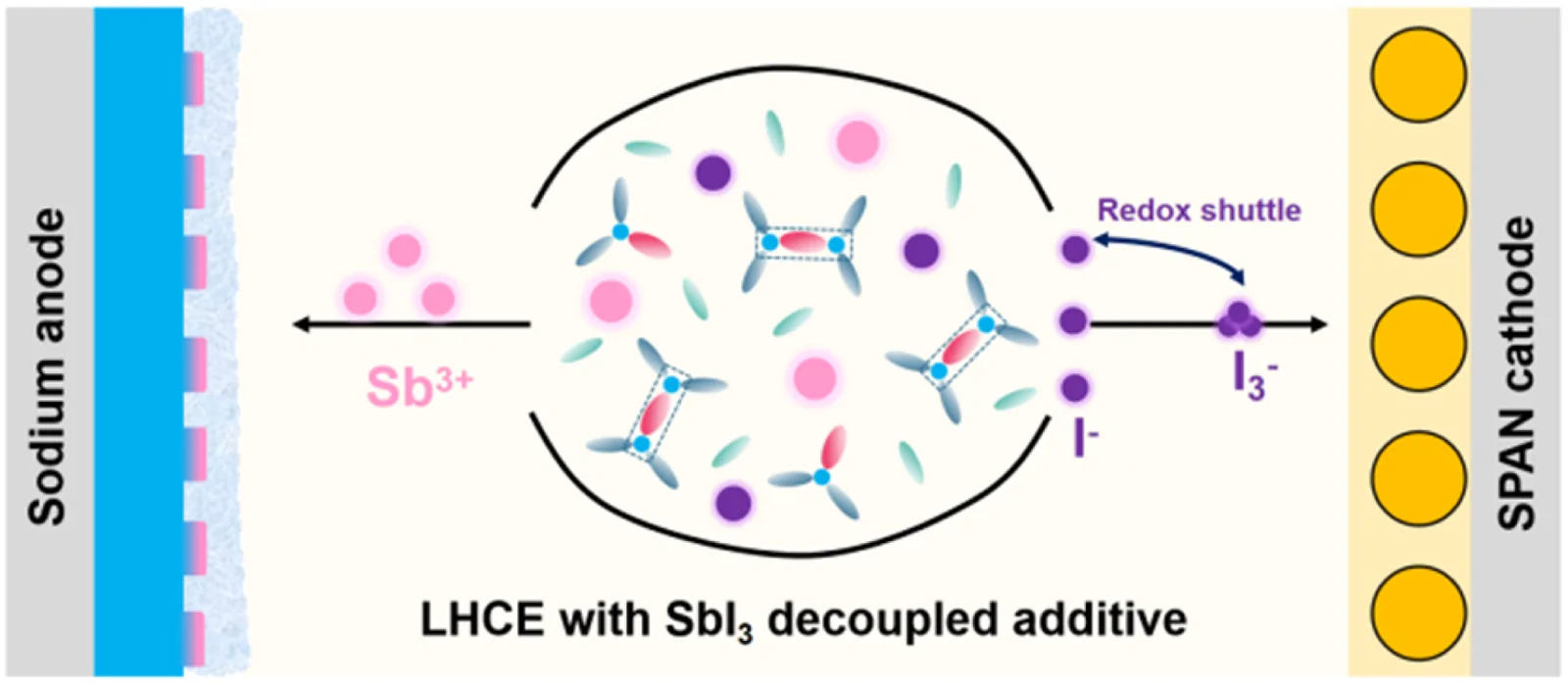

## 1. Introduction

Room-temperature sodium–sulfur (RT Na–S) batteries have attracted increasing interest for next-generation large-scale energy storage, owing to the natural abundance, low cost, and environmental friendliness of both sodium and sulfur, combined with a high theoretical energy density of 1274 W h kg^−1^.^1–3^ However, their practical viability is severely hindered by two interrelated challenges, the notorious shuttle effect of polysulfides (NaPSs) from sulfur conversion and the unstable sodium anode interface, which together form a deleterious feedback loop that accelerates capacity decay, lowers coulombic efficiency, and raises safety concerns.^4–6^

To address the radical challenges, extensive efforts have been devoted to electrolyte engineering. Localized high-concentration electrolytes (LHCEs) have recently emerged as a promising strategy to suppress polysulfide dissolution and dendrite growth. By combining a high concentration of sodium salt with a weakly solvating diluent, LHCEs achieve an anion-rich solvation structure dominated by contact ion pairs (CIPs) and aggregates (AGGs), with minimal free solvent molecules.^7–9^ This unique solvation environment can dramatically reduce the solubility of NaPS intermediates and promote the formation of an inorganic-rich solid-electrolyte interphase (SEI) on the sodium anode through preferential anion decomposition. Nevertheless, the fundamental limitation of relatively low ionic conductivity of LHCEs remains inadequately addressed and tends to sacrifice the rate capability for cycling stability.^10–12^ More critically, LHCEs address the shuttle effect of polysulfides through lowering their dissolution in electrolyte but do not eliminate their formation.^13–15^

A more fundamental solution lies in redesigning the sulfur cathode to intrinsically avoid soluble intermediate formation. Sulfurized polyacrylonitrile (SPAN) has emerged as a compelling alternative to elemental sulfur. Unlike elemental sulfur, which relies on the dissolution–precipitation mechanism with soluble NaPS intermediates, SPAN features covalently bonded short-chain sulfur embedded in a conductive carbon matrix, enabling a quasi-solid-state conversion that inherently suppresses polysulfide dissolution and shuttling.^16–20^ However, this solid-state reaction pathway comes at the cost of sluggish redox kinetics and poor reversibility, owing to the limited ionic/electronic transport and high energy barriers for S–S bond cleavage and reformation.^21–24^ Therefore, activating the SPAN cathode through efficient mediators or structural modification is essential to unlock its full potential.

In this context, electrolyte additives have been recognized as a versatile approach to realize specific functionalities without altering electrolyte structures. To date, however, most additives have been developed to tackle either the Na anode or the S cathode in isolation, including sodiophilic additives for Na deposition or redox mediators for sulfur conversion.^25–29^ Thus, it is ideal to design a dual-functional additive that simultaneously and independently benefits both electrodes.

Herein, we introduce antimony triiodide (SbI_3_) as a decoupled electrolyte additive into an LHCE for RT Na–S batteries with SPAN cathodes. We demonstrate that the SbI_3_ serves two spatially independent yet synergistic functions (Fig. 1). On the anode side, Sb species induce a sodiophilic Na_3_Sb interphase, which lowers the nucleation overpotential and guides more uniform sodium deposition. On the sulfur cathode side, the iodide redox mediator can attack the unsaturated C≡N bonds with high electron density in the SPAN framework *via* the spontaneously formed I_3_^−^. The formed polar C–I bonds in the SPAN skeleton can perturb the adjacent C–S bonds and weaken the S–S linkages within the –C–S_*x*_–C– network, thereby accelerating the quasi-solid-state redox kinetics and improving reaction reversibility. Consequently, the RT Na-SPAN batteries with the SbI_3_-modified LHCE deliver significantly improved rate capability and cycling stability.

**Fig. 1:**
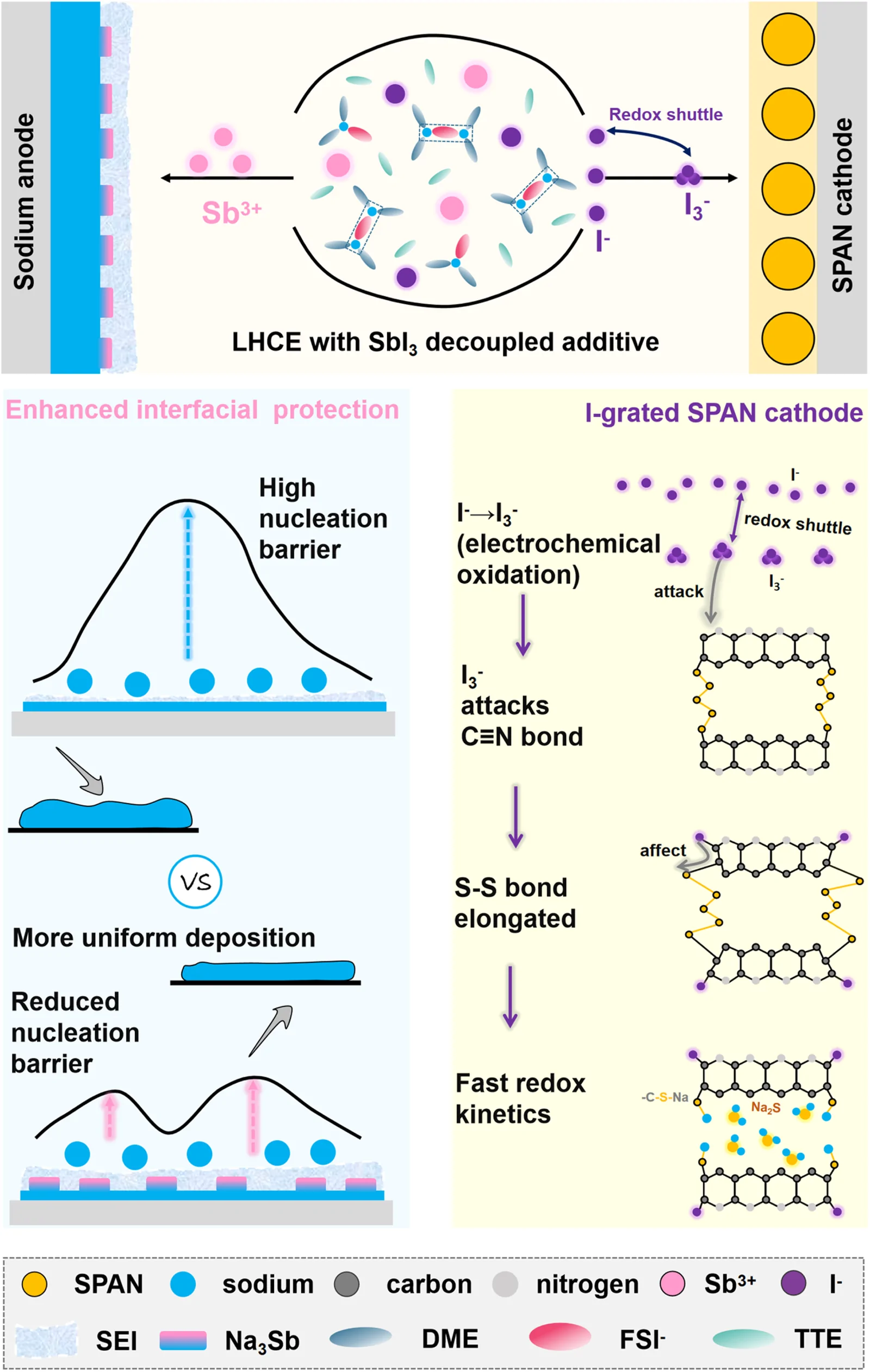
Schematic illustration of the decoupled bifunctional mechanism of the SbI_3_ additive in RT Na–S batteries.

## 2. Results and discussion

### 2.1 Design and characterization of electrolyte

For LHCEs, the incorporation of an additive must preserve their intrinsic solvation structures, ensure the critical roles of decreasing polysulfide dissolution and suppressing dendritic sodium, and additionally endow the electrolyte with targeted functionalities. A classic LHCE is employed in this work, which is composed of sodium bis(fluorosulfonyl)imide (NaFSI), 1,2-dimethoxyethane (DME), and 1,1,2,2-tetrafluoroethyl 2,2,3,3-tetrafluoropropyl ether (TTE) with a molar ratio of 1.0 : 1.2 : 1.0. The selected SbI_3_ additive has a very low solubility in the LHCE, so it can naturally be added in trace amounts to the electrolyte. As shown in Fig. S1, the LHCE with a trace amount of SbI_3_ turns red but remains transparent.

Molecular dynamics simulations were firstly carried out to understand the structural changes of the electrolyte after using the SbI_3_ additive. The radial distribution functions (RDFs) in Fig. 2b and c suggest that the two electrolytes show identical coordination trends of sodium ions. The coordination number statistics in Fig. 2d quantitatively indicate that the coordination number of FSI^−^ and DME around Na^+^ is approximately 3.5 and 1.5 in the LHCE electrolyte and that the coordination number of FSI^−^ and DME changes to 3.8 and 1.2 in the LHCE–SbI_3_ electrolyte. Fig. 2a illustrates typical molecular configurations of the Na^+^ solvation sheath in different electrolyte systems to deepen the understanding of the changes in sodium ion coordination structures at an atomic level. In dilute electrolyte (DE) with 1 M NaFSI in DME, Na^+^ is predominantly surrounded by DME solvent molecules, forming a solvent-dominated solvation sheath with limited anion participation. In high-concentration electrolyte (HCE), Na^+^ and FSI^−^ anions form compact CIP/AGG structures, with a reduced number of solvent molecules and a significantly increased proportion of anion coordination. In the LHCE, the introduction of the inert diluent TTE preserves the anion-dominated coordination structure. The introduction of SbI_3_ further strengthens the coordination between anions (FSI^−^) and Na^+^, forming more compact AGG structures, and the SbI_3_ stabilizes the solvation sheath through interactions with FSI^−^ or Na^+^, thereby reducing the number of free solvent molecules.

**Fig. 2:**
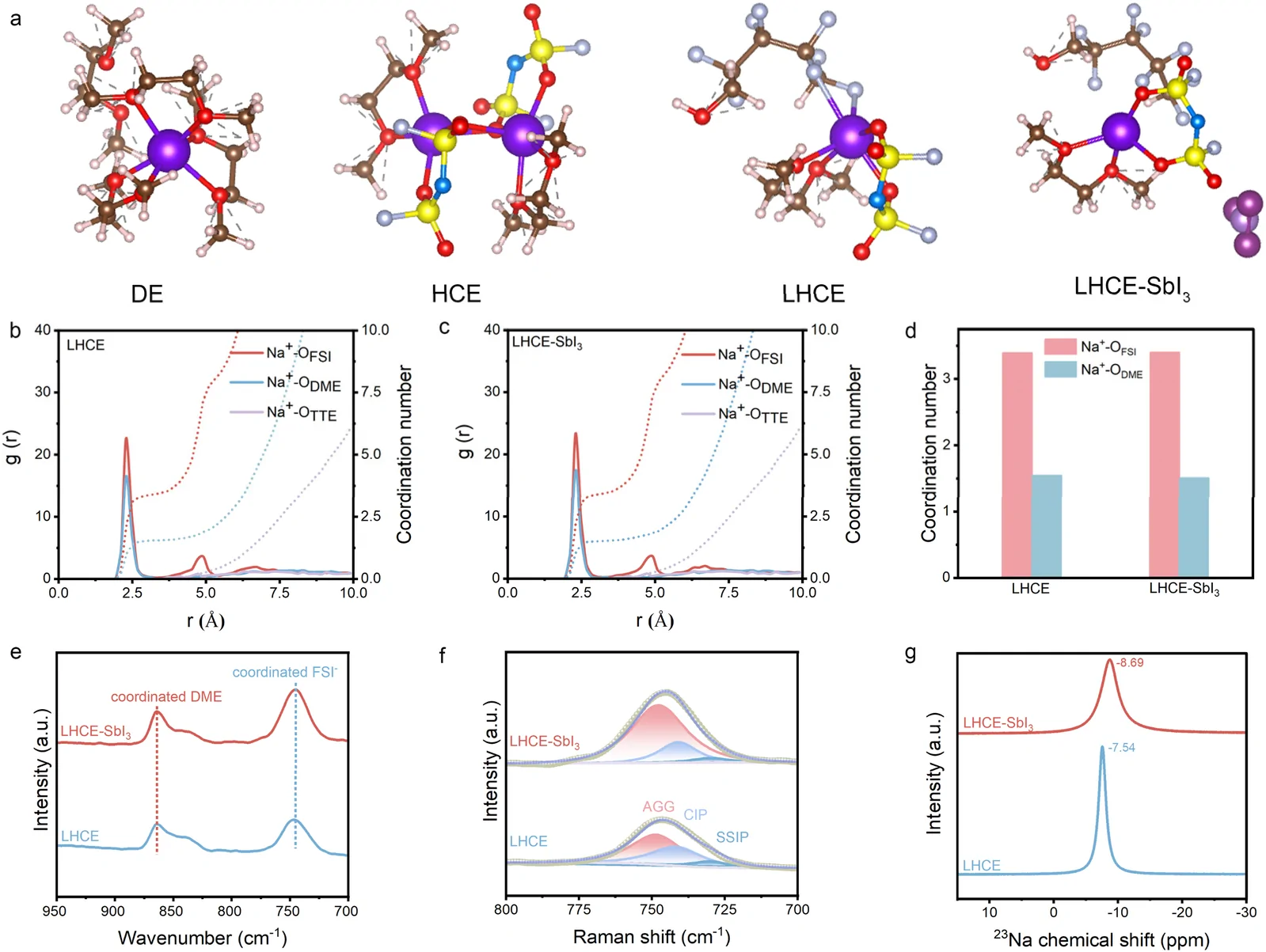
(a) Schematic illustration of the molecular configurations of DE, HCE, LHCE, and LHCE–SbI_3_. RDFs derived from MD simulations for (b) LHCE and (c) LHCE–SbI_3_ electrolyte. (d) Average coordination numbers of Na^+^ with FSI^−^ anions and DME solvent molecules in LHCE and LHCE–SbI_3_ electrolytes. (e) and (f) Raman spectra and corresponding peak deconvolution of LHCE and LHCE–SbI_3_ electrolytes. (g) ^23^Na NMR spectra of LHCE and LHCE–SbI_3_ electrolytes.

Fig. 2e displays the Raman spectra of the LHCE before and after incorporating the additive. In contrast to the pure DME (Fig. S2), Raman spectra of the electrolyte with/without the SbI_3_ additive exhibit two strong peaks, ascribed to coordinated DME and FSI^−^ anions, separately, indicating that the additive cannot affect the total coordination of sodium ions.^30^Fig. 2f further shows the changes in solvation structures induced by the SbI_3_ additive. The SbI_3_ promotes the formation of AGGs and reduces CIPs and solvent-separated ion pairs (SSIPs). Fig. S3 quantitatively determines the distinct fractions of three coordination structures in the electrolyte. The LHCE contains 7.85% SSIPs, 35.50% CIPs, and 56.65% AGGs, and for the LHCE–SbI_3_, the proportion of AGGs is significantly increased to 77.23%, while the SSIP fraction decreases to 3.96% and the CIP fraction to 18.81%. Furthermore, the coordination strength between Na^+^ ions and FSI^−^ anions in the solvation structure was probed by ^23^Na nuclear magnetic resonance (NMR) spectroscopy. As shown in Fig. 2g, the peak undergoes an upfield shift upon the addition of SbI_3_. This indicates that the introduction of SbI_3_ strengthens the interaction between Na^+^ ions and FSI^−^ anions, leading to increased electron density on the Na^+^ ions and an enhanced shielding effect. Consequently, the addition of SbI_3_ can induce localized high-concentration behavior in the electrolyte, resulting in a tunable solvation structure of Na^+^–FSI^−^ complexes. We further measured the ionic conductivities of the LHCE and LHCE–SbI_3_ electrolytes at room temperature. The LHCE exhibits an ionic conductivity of 1.928 mS cm^−1^, while the LHCE–SbI_3_ shows a slightly decreased value of 1.923 mS cm^−1^. This indicates that the introduction of SbI_3_ does not compromise the ionic transport properties of the LHCE; instead, the optimized solvation structure facilitates efficient Na^+^ transport. Linear sweep voltammetry in Fig. S4 also reveals that the use of additive does not alter the electrochemical stability of the LHCE.

The aforementioned results fully demonstrate that the addition of SbI_3_ does not disrupt the characteristic solvation structures of the LHCE but rather optimizes it to produce more AGGs. This will have great influence on the interphase chemistry at the electrolyte/electrode interfaces.

### 2.2 The role of additive in the sodium anode

To begin with, the morphologies of sodium metal after 20 h of stripping/plating in different electrolytes were analyzed by scanning electron microscopy (SEM). As shown in Fig. S5, the sodium metal exhibited a smoother and denser plated morphology in the case of LHCE–SbI_3_. To investigate the stability of the electrolyte/electrode interface, electrochemical impedance spectroscopy (EIS) was conducted on Na‖Na symmetric cells after standing for 20 h (Fig. S6). The LHCE–SbI_3_ cell displayed a much lower interfacial resistance than that with the LHCE. These results demonstrate that the additive can effectively induce denser and more stable interface layers on the sodium anode.

To further elucidate the mechanistic role of the SbI_3_ additive in the sodium anode, X-ray photoelectron spectroscopy (XPS) was conducted on different etching depths to analyze the chemical composition and distribution of the SEI. Fig. S7 shows the full XPS spectra of the sodium anode with the two electrolytes after cycling. The C 1s spectra in both Fig. 3a and d display three peaks at 284.8, 288.6, and 289.4 eV, corresponding to the formation of C–C, C–O, and C=O bonds, respectively.^31–33^ In contrast, the sodium anode with the SbI_3_ additive exhibits substantially higher C–C bond content after 50 s and 100 s of etching, with an intense C–C peak already dominant at the surface, while the C–O/C=O peak intensities remain extremely low. This demonstrates that SbI_3_ effectively suppresses excessive solvent decomposition to generate less organic components within the SEI, resulting in a thinner inorganic-rich interfacial layer on the sodium anode.

**Fig. 3:**
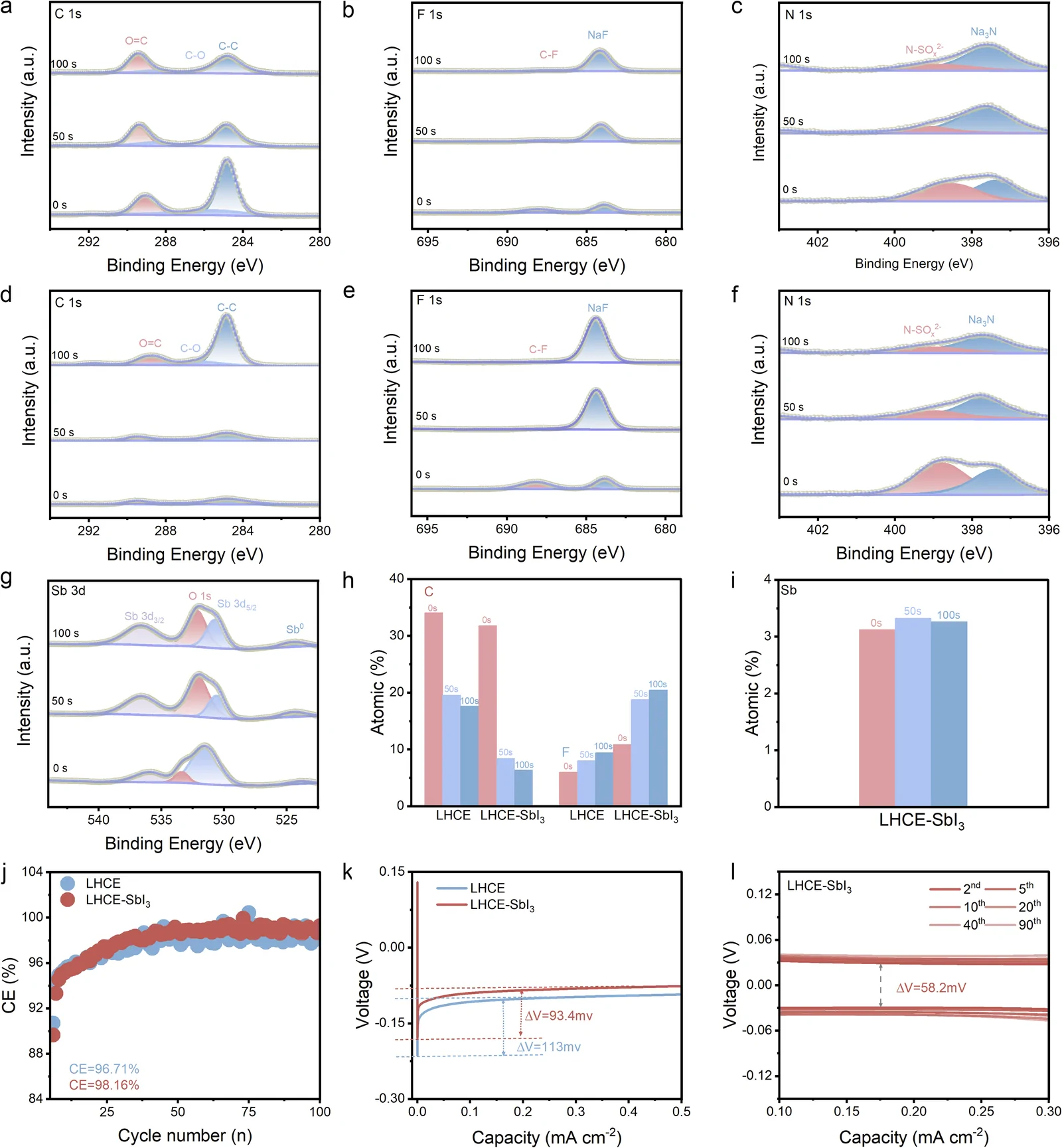
High-resolution XPS spectra of the sodium anode surface after cycling in the LHCE electrolyte: (a) C 1s, (b) F 1s, and (c) N 1s. High-resolution XPS spectra of the sodium anode surface after cycling in the LHCE–SbI_3_ electrolyte: (d) C 1s, (e) F 1s, and (f) N 1s. (g) High-resolution Sb 3d XPS spectrum of the sodium anode surface after cycling in the LHCE–SbI_3_ electrolyte. (h) Atomic proportion of elemental C and F in the SEI layer at different etching time. (i) Atomic proportion of Sb element at different etching time in the SEI layer. (j) and (k) CE and corresponding initial voltage–capacity profile of Na‖Cu half cells employing LHCE and LHCE–SbI_3_ electrolytes. (l) Voltage–capacity profiles of a Na‖Cu half cell with the LHCE–SbI_3_ electrolyte at selected cycles.

In the F 1s spectra in Fig. 3b and e, the NaF signal is significantly enhanced when adopting the additive, and its intensity continues to increase with longer etching time. This indicates that the additive induces the formation of a NaF-rich inorganic SEI layer which can reduce interfacial impedance and enhance mechanical robustness. The N 1s spectra in Fig. 3c and f further reveal differences in the distribution of Na_3_N. The sample exhibits an obviously enhanced Na_3_N peak at 397.7 eV when using the additive. As an inorganic species with high ionic conductivity, the enrichment of Na_3_N is able to improve the ion transport capability within the SEI.^34,35^ Moreover, the intensity of the N–SO_*x*_^2−^ peak (398.9 eV) originating from salt decomposition is significantly reduced in comparison to the additive-free case. This reflects the great inhibition effect of the additive on parasitic side reactions on electrode surfaces.

It is worth noting that for the sample using the additive, no signal ascribed to I element is detected but the signal of Sb element is successfully collected. This indicates that the Sb element exerts greater influence on the chemical composition of anode interphase layers than I element. Fig. 3g presents the XPS spectra of Sb 3d at different depths. The Sb 3d peaks (Sb 3d_5/2_ and Sb 3d_3/2_) always exist at various etching depths, and their intensities are relatively stable with increasing etching depth. This means that Sb species are uniformly distributed throughout the SEI layer. It is of greater importance that the Sb^0^ generated during the electrochemical process can naturally react with sodium ions to form the highly sodiophilic Na_3_Sb. The alloying reaction between Sb^0^ and sodium ions can be expressed as 2Sb + 6Na^+^ + 6e^−^ → 2Na_3_Sb. Although the possible presence of residual intermediate Sb species (*e.g.*, Sb^3+^ or Sb_2_O_3_) cannot be completely excluded, the overall reducing environment during electrochemical cycling favors the formation of metallic Sb and its subsequent alloying with sodium. The previous studies have proved that these Sb-contained components within SEI layers are highly sodiophilic and conducive to reducing the sodium nucleation barrier as well as guiding uniform and ordered sodium deposition.^36–38^

Fig. 3h and i presents the atomic proportion changes of SEI layers with varied etching time. It directly reveals the gradient variation of chemical components within SEI layers and also confirms the additive-induced richer inorganic components on sodium anode surfaces as well as the uniformly distributed Sb-contained species within the SEI layers. Certainly, this also verifies that the decomposition products of Sb^3+^ participate integrally in the construction of the SEI film rather than merely adsorbing on the surface.

Next, the influence of the electrolyte additive on sodium deposition is comprehensively evaluated through assembling Na‖Cu half cells and Na‖Na symmetric cells. Fig. 3j presents the long-term cycling CE of Na‖Cu half cells employing the LHCE and LHCE–SbI_3_ electrolytes. Upon the introduction of the SbI_3_ additive, the average CE reaches 98.16%, notably surpassing the 96.71% attained with the LHCE. The significantly enhanced average CE exhibits superior stability throughout prolonged cycling. The initial voltage–capacity profiles under the two electrolytes exhibit different voltage hysteresis, as shown in Fig. 3k. Thereinto, the cell with the LHCE–SbI_3_ displays a polarization voltage of 93.4 mV, whereas the LHCE counterpart exhibits a considerably larger polarization voltage of 113 mV. Such a contrast directly signifies the sodium nucleation barrier when using the LHCE–SbI_3_.^39–41^Fig. 3l illustrates the evolution of the voltage profiles of the LHCE–SbI_3_ system at selected cycle numbers. Even after 90 cycles, the polarization voltage only increases to 58.2 mV, far lower than the 71.5 mV observed in the Na‖Cu cell with the baseline LHCE (Fig. S8). Besides, the plating/stripping plateaus remain flat after long-term cycling, with no discernible voltage fluctuation or hysteresis escalation. This fully demonstrates that the SEI layer constructed by this additive possesses excellent mechanical robustness and chemical stability, capable of accommodating the volumetric strains during sodium plating/stripping and preventing a sharp rise in interfacial impedance with prolonged cycling.

Fig. S9a presents the voltage response of Na‖Na symmetric cells with the two electrolytes under long-term galvanostatic cycling. The symmetric cell assembled with the LHCE–SbI_3_ exhibits an extremely low overpotential and a very stable voltage profile over a test duration exceeding 1100 hours. The enlarged view further in Fig. S9b reveals that the voltage fluctuation in the LHCE–SbI_3_ system is minimal, whereas the LHCE system displays gradually increasing overpotential and unstable oscillations. This result robustly confirms that the SbI_3_ additive effectively guides uniform sodium deposition, suppresses dendrite growth, and thus ensures the safety and stability of the sodium metal anode under long-term operation.

The abovementioned morphological observation, chemical composition analysis, and electrochemical test results consistently demonstrate that the SbI_3_ additive reduces the sodium nucleation barrier, decreases interfacial impedance and voltage polarization, and enhances the cycling stability through the *in situ* construction of a robust inorganic-richer SEI layer.

### 2.3 The iodine-regulated sulfur redox kinetics

The focus is shifted to the influence of additive on the quasi-solid-state sulfur conversion reactions. Previous studies have conclusively demonstrated that metal elements such as Bi, In, and Sb in electrolyte do not exert a significant influence on the redox chemistry of sulfur, so the emphasis is placed on the I^−^-mediated quasi-solid-state sulfur redox kinetics.^27,36^ To unravel the influence of the iodine redox mediator on the electrochemical kinetics of the SPAN cathode, we performed a comprehensive analysis of key intermediates and final products of the reaction using time-of-flight secondary ion mass spectrometry (TOF-SIMS).

First, the uniformly distributed I^−^ signal in Fig. 4a confirms that iodine atoms are not present as elemental iodine or in a physically adsorbed state, but rather form stable C–I covalent bonds *via* electrophilic attack on the unsaturated carbon sites of the SPAN backbone, specifically the C≡N bonds in cyclized PAN (Fig. S10a). This covalent bonding state provides the structural basis for affecting sulfur redox reactions. The S^−^ signal, a characteristic fragment of the C–S bonds in the SPAN skeleton, shows uniform distribution and high intensity within the SPAN skeleton (Fig. 4b), indicating that iodine atoms do not disrupt the intrinsic C–S conjugated framework. These results confirm that iodine incorporation is a backbone-grafting modification rather than a replacement or destruction of the sulfur active centers.

**Fig. 4:**
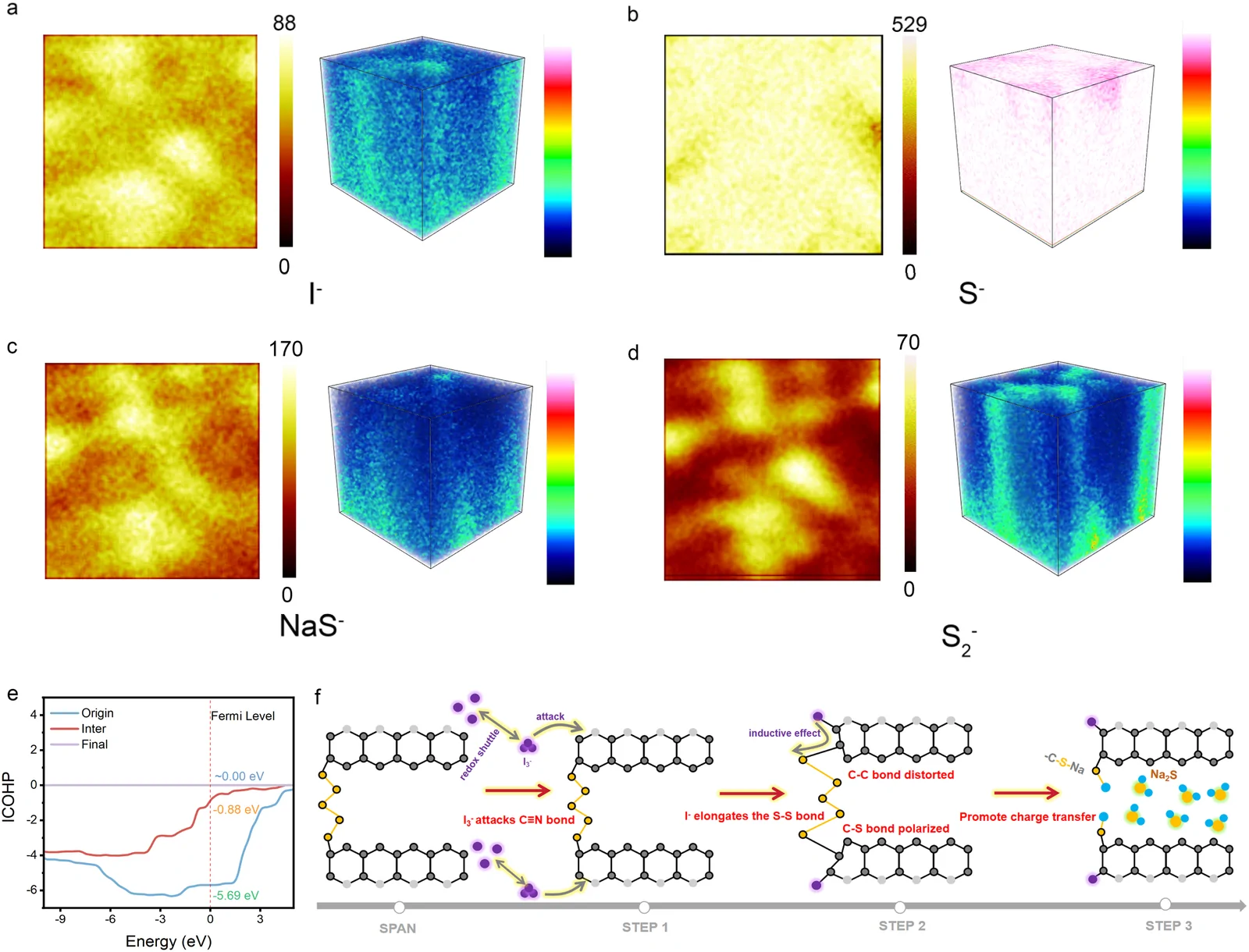
(a)–(d) 2D and 3D reconstructed TOF-SIMS mapping of characteristic ion fragments for the SPAN cathode after cycling. (e) The iCOHP descriptor for the S–S bonds in the SPAN. (f) Schematic illustration of *in situ* iodine grafting-induced S–S bond cleavage and accelerated sulfur redox reactions during the discharge process.

At the reaction-pathway level, fragment analysis of the discharge products reveals a unique solid–solid conversion behavior in this system. As shown in Fig. 4c and d, the ion fragments of NaS^−^ and S_2_^−^ (assigned to Na_2_S and Na_2_S_4_, respectively) exhibit relatively weak signal intensities and sparse distributions. Although the complete absence of polysulfide species cannot be rigorously guaranteed solely by the detection limits of TOF-SIMS, the substantially suppressed S_2_^−^ signal, combined with the high reversibility and low polarization observed in electrochemical tests, strongly suggests that the iodine-grafted SPAN cathode promotes more complete solid-state sulfur conversion while minimizing polysulfide accumulation. This observation is consistent with the accelerated redox kinetics enabled by the weakened S–S bonds discussed above. In addition, the uniformly distributed F^−^ and the relatively strong CO_3_^−^ fragments detected by TOF-SIMS (Fig. S14b and c) jointly reveal the formation of a gradient CEI film derived from anion decomposition in the LHCE. This inorganic-dominant interfacial layer can facilitate rapid Na^+^ transport in quasi-solid-state sulfur redox reactions.

Theoretical calculations were further conducted to understand the influence of the iodine redox mediator on sulfur redox reactions (Fig. 4e). Here, we propose the integrated crystal orbital Hamilton population (iCOHP) as a descriptor to understand the interaction process between SPAN and I^−^. At the initial state, an integrated bonding state value of −5.96 eV is observed, indicating that the S–S bond in SPAN is difficult to break. After introducing iodine into the reaction system, the bonding state value is significantly altered to −0.88 eV. This indicates that the iodine can greatly promote the cleavage of S–S bonds within the SPAN skeleton and thus accelerate sulfur redox reactions.

Based on the previous studies and findings in this work, a redox mechanism of the SPAN cathode mediated by iodine is proposed for RT Na–S batteries. Fig. 4f schematically presents the molecular mechanism of quasi-solid-state reactions for SPAN mediated by iodine. At the early stage of discharge, the free I^−^ ions in the electrolyte aggregate into I_3_^−^, which then attacks the unsaturated carbon atoms of the C≡N bond within the SPAN skeleton *via* an electrophilic attack pathway. This forms a stable C–I covalent bond in the SPAN skeleton, disrupts the original C≡N conjugated structure, and induces distortion of the adjacent C–C bonds. After the inductive polarization is accomplished, the C–I bond remains stable, while the I^−^ and I_3_^−^ ions near the interface exhibit an extremely fast electron self-exchange rate, allowing the I_3_^−^ to be reduced back to I^−^. The re-accumulated I^−^ is then converted again into I_3_^−^, and this continuous cycle ensures a sufficient supply of I_3_^−^ throughout the system, enabling sustained attack on unreacted C≡N sites. Consequently, only a trace amount of iodine doping is required to maintain the kinetics accelerating effect persistently. The strong electron-withdrawing inductive effect of the iodine atom in the C–I bond is directionally transmitted through the conjugated backbone to the neighboring chemical bonds. This effect firstly polarizes the electron cloud of the C–S bond that links the backbone and the sulfur chains, thereby weakening the charge density of the C–S bond. This polarization effect is further transmitted along the sulfur chain to the S–S bond, pulling its electron cloud away from the bond axis and effectively elongating and weakening the S–S bond. Once the S–S bond is sufficiently activated, Na ions can overcome the solid–solid interfacial energy barrier and intercalate into the SPAN skeleton. The polarized and weakened S–S bonds rapidly cleave during this process and combine with Na ions to form a –C–S–Na intermediate, which eventually converts to the Na_2_S discharge product. Meanwhile, the strain energy stored in the C–C backbone is released upon S–S bond cleavage, allowing the pyridine ring to undergo structural relaxation and return to its normal configuration. Throughout this entire process, the polarization effect induced by the C–I bond continuously reduces the charge-transfer resistance at the solid–solid interface, enabling rapid electron/ion transport across the interface and efficient sulfur reduction reaction.

Based on the C–I covalent grafting mechanism and the unique quasi-solid-state conversion pathway, we further quantitatively evaluated the enhancement effect of iodine grafting on sulfur redox kinetics through systematic electrochemical analysis. The SPAN cathode was selected to pair with a sodium anode and assemble Na–S batteries. The cyclic voltammetry (CV) measurements were performed at a scan rate range from 0.2 to 0.6 mV s^−1^. The cells employing the two electrolytes possess the same redox peaks. The reduction peaks are located at approximately 1.87 V and 1.07 V, while the oxidation peaks appear at around 1.46 V and 1.99 V (Fig. S11). These identical peak potentials quantitatively confirm that the two electrolyte systems share the same redox mechanism.

To quantitatively assess the influence of iodide on the sulfur redox kinetics, the sodium-ion diffusion coefficient (*D*_Na^+^_) was further calculated using the following equation:*I*_p_ = 2.65 × 10^5^*n*^1.5^*SD*_Na^+^_^0.5^*Cν*^0.5^

In this equation, *I* denotes the peak current, *n* represents the number of transferred electrons, *S* is the electrode area, *C* is the sodium-ion concentration, and *ν* is the scan rate. It can be inferred that a larger slope of the fitted curve corresponds to a higher sodium-ion diffusion coefficient, further reflecting sulfur conversion kinetics. The cell employing LHCE–SbI_3_ exhibits a higher slope than that with the LHCE counterpart, confirming that I^−^ indeed facilitates quasi-solid-state sulfur redox kinetics (Fig. 5a and b). Fig. S12 presents the evolution of the sodium-ion diffusion coefficient measured by the galvanostatic intermittent titration technique (GITT). The overall polarization of the Na–S cell using the LHCE–SbI_3_ is significantly reduced, with diminished voltage hysteresis between the charge and discharge steps. Moreover, the LHCE–SbI_3_ system displays higher *D*_Na^+^_ values than the LHCE system almost throughout the entire charge/discharge process, particularly in the discharge process (Fig. 5c). This confirms that the I^−^ redox mediator constructs a low-impedance interfacial layer to effectively reduce the solid-phase diffusion barrier of sodium ions within the SPAN electrode. EIS in Fig. 5d further manifests that the charge-transfer resistance of the cell with LHCE–SbI_3_ is lower than that of the LHCE counterpart.

**Fig. 5:**
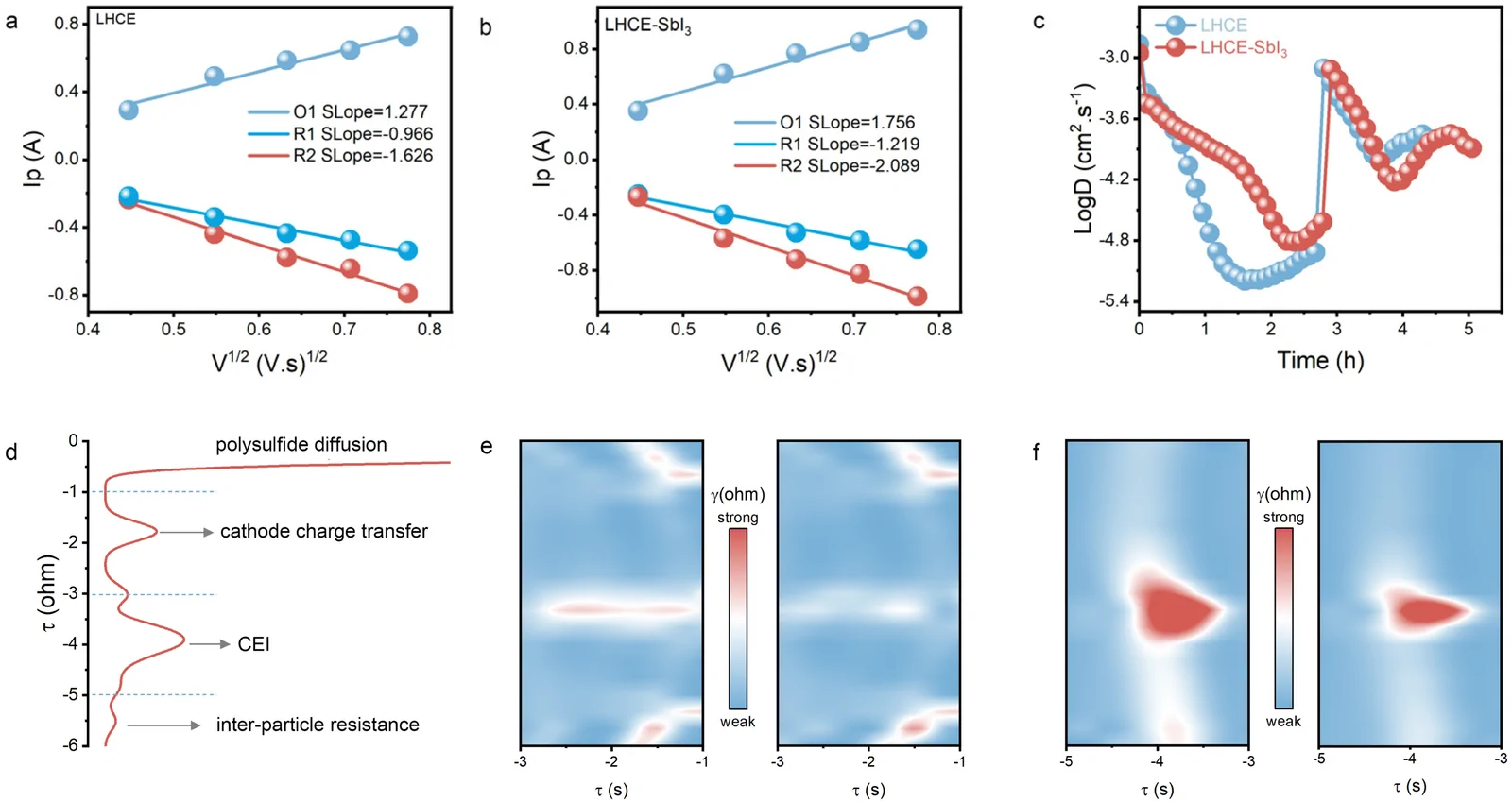
(a) and (b) Linear fitting curves of the peak current (*I*) *versus* the square root of the scan rate (*ν*^1/2^) for Na–S cells employing LHCE and LHCE–SbI_3_ electrolyte. (c) Sodium-ion diffusion coefficient calculated from GITT measurements. (d) The DRT spectra derived from the initial EIS plots of the cells with LHCE–SbI_3_ electrolyte. (e) and (f) Contour maps obtained from DRT spectra derived from the *in situ* EIS data. The left represents the LHCE, and the right is the LHCE–SbI_3_.

To evaluate sodium-ion transport and polysulfide diffusion kinetics during dynamic reaction processes, *in situ* EIS using the two electrolytes during the discharge–charge process were recorded (Fig. S13). The corresponding interfacial dynamic process was decoupled with the distribution of relaxation time (DRT). Fig. S14 displays the DRT spectrum of cells with the two electrolytes measured at open-circuit voltage. These characteristic peaks reflect different electrochemical processes, including polysulfide diffusion impedance, cathode–electrolyte interphase (CEI) resistance, cathode charge transfer resistance, and inter-particle resistance.^42–44^Fig. 5e and f identify the impedance components corresponding to various regions. It is clearly evident that both the cathode charge transfer resistance and the CEI resistance of the Na–S cell employing LHCE–SbI_3_ are substantially lower than those of the LHCE counterpart. This pronounced contrast demonstrates that LHCE–SbI_3_ endows the cell with superior sodium-ion conductivity, establishing the foundation for superior sulfur redox kinetics.

### 2.4 Electrochemical performance evaluation

Based on the aforementioned discussion, the proposed decoupled electrolyte additive can realize the bifunctionality of reducing the sodium nucleation barrier and accelerating sulfur conversion kinetics through Sb^3+^ decomposition and I^−^ mediation, which is expected to contribute to durable and high-rate RT Na–S batteries. As such, the electrochemical performance is comprehensively evaluated to validate the enhancement effect of the decoupled bifunctional electrolyte additive on Na–S electrochemistry.

As displayed in Fig. 6a, the LHCE–SbI_3_ cell can maintain higher capacity than the LHCE counterpart throughout 300 cycles at 0.2C. Fig. S15 shows their discharge–charge profiles at selected cycles. The much higher capacity fully indicates the stronger reaction activity of the sulfur cathode induced by the decoupled additive. Fig. 6b summarizes reported literature about Na-SPAN batteries to compare their cycling performance at a small rate. This work achieves a record in capacity, demonstrating the effectiveness of the as-designed electrolyte in RT Na–S batteries. Even when the sulfur loading is increased to 2 mg cm^−2^, the Na–S cell with the LHCE–SbI_3_ can still obtain a high specific capacity over 500 mA h g^−1^ after 200 cycles at 0.5C (Fig. S16).

**Fig. 6:**
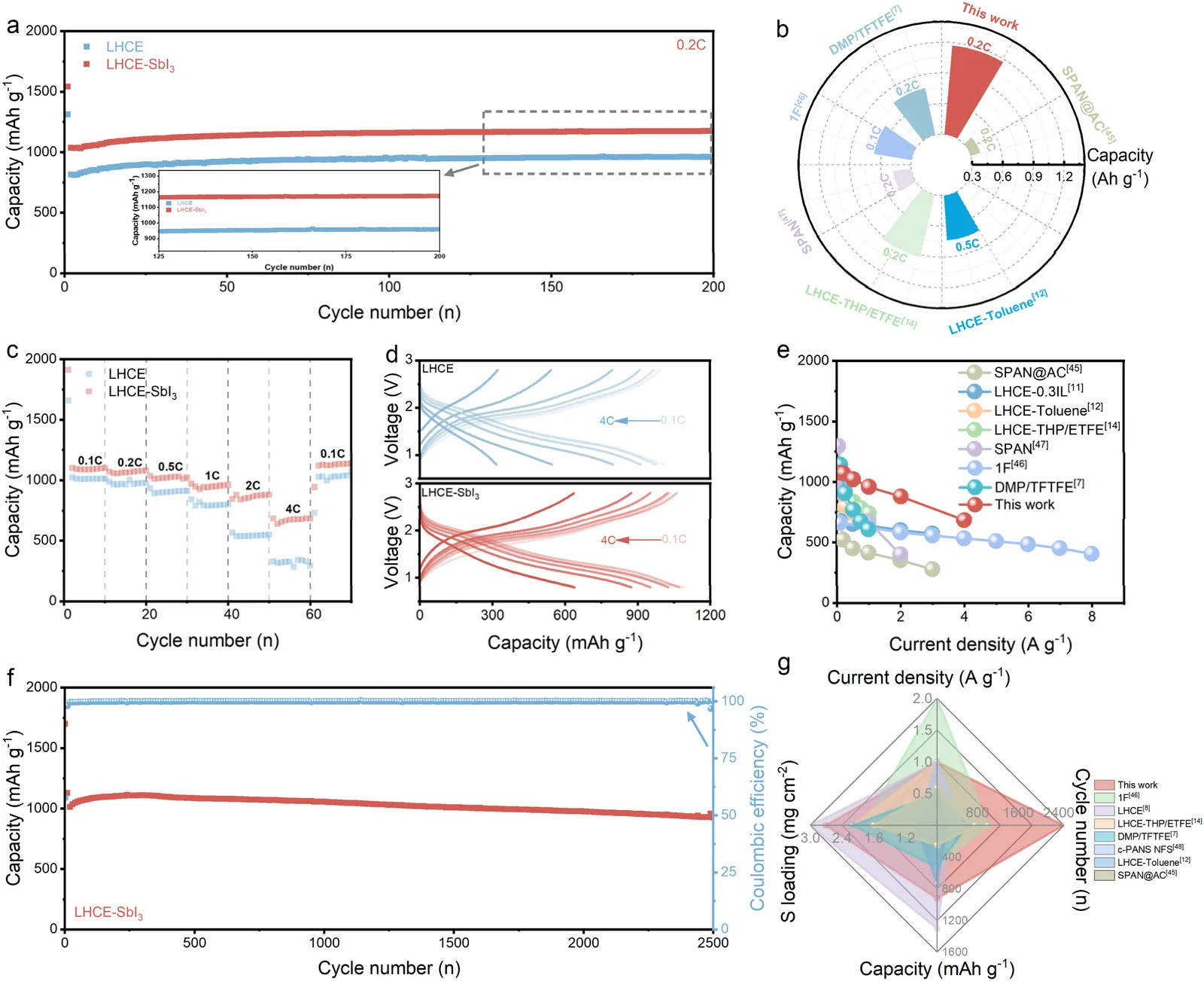
(a) Short-term cycling performance comparison of RT Na–S full cells at 0.2C. (b) Capacity comparison between this work and other reported RT Na–S batteries at low rates.^7,12,14,45–47^ (c) Rate performance of Na–S full cells at various rates ranging from 0.1C to 4C. (d) Discharge–charge profiles of Na–S full cells at different rates. (e) Rate performance comparison between this work and other reported Na–S batteries.^7,11,12,14,45–47^ (f) Long-term cycling stability of an Na–S full cell employing the LHCE–SbI_3_ electrolyte at 1C. (g) Comprehensive comparison between this work and other reported Na–S batteries with respect to sulfur loading, specific capacity, cycle number, and current density.^7,8,12,14,45,46,48^

The rate capability of Na-SPAN cells with the LHCE and LHCE–SbI_3_ electrolytes was also obtained, as displayed in Fig. 6c. Reversible capacities of 1075, 1071, 1023, 960, 882, and 685 mA h g^−1^ are achieved by the Na–S cell with the LHCE–SbI_3_ at rates of 0.1, 0.2, 0.5, 1, 2, and 4C, respectively, which are much higher than those of the LHCE counterpart. When the C-rate is returned to 0.1C, it can even recover a high capacity of 1122 mA h g^−1^, exceeding the initial capacity. This is due to the complete activation of active species during repeated cycling. The corresponding discharge–charge profiles in Fig. 6d further reveal that the polarization is substantially smaller for LHCE–SbI_3_ than that for the baseline LHCE, particularly at high rates of 2C and 4C, where the decrease in voltage polarization is even more pronounced. It is worth noting that the Na–S cell employing the LHCE–SbI_3_ can maintain well-defined voltage plateaus even at a rate of 2.0C. Fig. 6e further benchmarks the rate performance of this work against other LHCE-based Na–S batteries, corroborating the significant improvement in sulfur reaction kinetics through the rational design of an additive for LHCEs.

Furthermore, to stabilize the electrode/electrolyte interfaces and ensure a consistent starting state before high-rate evaluation, the Na–S cell employing the LHCE–SbI_3_ was pre-cycled for 10 cycles at 0.2C (Fig. S17), followed by a long-term cycling test at a high rate of 1.0C (Fig. 6f), with a SPAN loading of 2.71 mg cm^−2^ and an electrolyte-to-sulfur (E/S) ratio of 20 µL mg^−1^. It exhibits outstanding cycling stability over an ultra-long lifespan of 2500 cycles. To be specific, it achieves a capacity retention as high as 90.8%, corresponding to a capacity decay rate of only 0.09% per cycle. This fully validates the exceptional long-term stability and high reversibility of sulfur redox reactions with this electrolyte under relevant practical conditions. The radar chart in Fig. 6g demonstrates that this work exhibits significant competitive advantages over other reported systems across multiple dimensions, including energy density, rate capability, and cycling lifespan.

## 3. Conclusion

In summary, this work demonstrates an *in situ* iodine grafting strategy on SPAN cathodes *via* a decoupled bifunctional SbI_3_ additive in an LHCE for durable and high-rate RT Na–S batteries. The Sb^3+^ forms a sodiophilic Na_3_Sb interphase that lowers the Na nucleation overpotential, while the iodine grafting weakens S–S bonds in SPAN and accelerates quasi-solid-state redox kinetics. The synergistic interplay between LHCE and the decoupled additive delivers outstanding cycling stability and rate capability. This work establishes a distinctive mediation mechanism for SPAN cathodes *via in situ* grafted iodine and offers a versatile design principle for high-performance metal–sulfur batteries.

## 4. Experimental section

### 4.1 Synthesis of SPAN

Sulfur powder and polyacrylonitrile (PAN) powder were blended at a mass ratio of 4 : 1. Specifically, 4 g of sulfur powder and 1 g of polyacrylonitrile powder were mixed under stirring at a rotation speed of 360 rpm for 10 h to achieve homogeneous mixing, yielding a yellow solid precursor powder. The as-prepared precursor was transferred to a tube furnace and thermally treated under an argon atmosphere. The heating rate was set to 10 °C min^−1^, and the sample was heated up to 450 °C with a holding time of 6 h. After natural cooling to room temperature, a black powder was collected and thoroughly ground to obtain the final sulfurized polyacrylonitrile (SPAN) composite. The sulfur content in the SPAN composite is determined to be 40 wt% *via* elemental analysis.

### 4.2 Synthesis of sulfur cathodes

SPAN, carbon nanotubes (CNTs) and polyvinylidene fluoride (PVDF) were mixed at a mass ratio of 7 : 2 : 1 and dispersed in *N*-methyl-2-pyrrolidone (NMP) solvent, followed by grinding to yield a homogeneous slurry. The as-obtained slurry was uniformly coated onto a carbon-coated aluminum foil current collector, and then dried overnight in a vacuum oven at 70 °C to fully evaporate the NMP solvent. The resultant electrode was subsequently punched into circular discs with a diameter of 10 mm, and the areal sulfur loading of each as-fabricated sulfur cathode was determined to be 0.9–1.1 mg cm^−2^.

### 4.3 Synthesis of electrolyte

Sodium bis(fluorosulfonyl)imide (NaFSI) was dried in a vacuum oven at 120 °C for 24 h prior to use. 1,2-Dimethoxyethane (DME) and 1,1,2,2-tetrafluoroethyl-2,2,3,3-tetrafluoropropyl ether (TTE) were dried over molecular sieves for 3 days before electrolyte preparation. All electrolyte formulations were carried out in an argon-filled glovebox with both O_2_ and H_2_O concentrations maintained below 0.01 ppm. The localized high-concentration electrolyte (LHCE) was formulated with DME, NaFSI and TTE at a molar ratio of 1 : 1.2 : 1. To fabricate the modified localized high-concentration electrolyte, 1 wt% of SbI_3_ was introduced as a functional additive into the as-prepared LHCE. The mixture was stirred at room temperature for 12 h to ensure complete dissolution of SbI_3_, and then allowed to stand for 24 h to remove undissolved precipitates. Finally, the supernatant clear solution was collected to yield the target modified localized high-concentration electrolyte.

### 4.4 Materials characterization

The morphologies of all as-prepared samples were characterized *via* scanning electron microscopy (SEM, Thermo Apreo 2S HiVac). X-ray photoelectron spectroscopy (XPS, Thermo Scientific ESCALAB 250Xi) was employed to analyze the chemical states of the samples. The sulfur content in SPAN was determined by thermogravimetric analysis (TG 209, Germany). A Raman spectrometer was utilized to analyze the structures of the electrolytes.

### 4.5 Electrochemical measurements

CR2032 coin cells were assembled with the as-prepared sulfur cathode discs in an argon-filled glovebox with both O_2_ and H_2_O concentrations maintained below 0.01 ppm. The sodium foil employed had a diameter of 10 mm and a thickness of 500 µm. A glass fiber separator (Whatman, GF/A) with a diameter of 16 mm was adopted as the cell separator. A volume of 40 µL of electrolyte was injected into each coin cell. Galvanostatic charge–discharge tests were performed on a LAND battery test system at room temperature within a voltage window of 0.8–2.8 V. Cyclic voltammetry (CV) curves were recorded on a Biologic VMP-3 electrochemical workstation.

### 4.6 Theoretical calculations

The bonding characteristics and fracture mechanisms of S–S bonds in SPAN are investigated *via* Crystal Orbital Hamilton Population (COHP) analysis. These calculations are implemented using the VASP package in conjunction with the LOBSTER software. VASP performs periodic density functional theory (DFT) calculations. LOBSTER projects the plane-wave basis set onto local orbitals to enable a rigorous definition of chemical bonding. This approach quantifies the strength of specific covalent interactions. Furthermore, the Bader charge analysis method evaluates the atomic charge distribution. This method utilizes the charge density output from VASP to determine the degree of electron transfer within the systems.

## Author contributions

M. Yang, X. Li, and Q. Feng: writing – review and editing, writing – original draft, validation, software, methodology, investigation, formal analysis, conceptualization. Y. Yang, X. Shen, and K. Chen: validation, supervision, software. L. Yao and K. Zhu: data acquisition, software, and formal analysis. X.-L. Huang, Z. Hu, H.-K. Liu, and Y.-X. Wang: methodology, funding acquisition, supervision, and writing – review and editing.

## Conflicts of interest

The authors declare no conflicts of interest.

## Supplementary Material

SC-017-D6SC06580E-s001

## Data Availability

The data that support the findings of this study are available from the corresponding author upon reasonable request. Supplementary information (SI) is available. See DOI: https://doi.org/10.1039/d6sc06580e.
