# Supplementary material for: In situ iodine-grafted SPAN via a decoupled additive for durable and high-rate Na–S batteries
Source: Chem Sci. 2026 Sep 18;17(42):20606–16. doi: 10.1039/d6sc06580e (PMC13588261; doi:10.1039/d6sc06580e)
Supplement: Supplementary file 1 [file SC-017-D6SC06580E-s001.pdf]

Supplementary Information (SI) for Chemical Science.
This journal is © The Royal Society of Chemistry 2026

Supporting Information

In-Situ Iodine-Grafted SPAN via a Bifunctional Additive for Durable and High-Rate Na-S Batteries

Mingyue Yang[§,1], Xue Li[2§], Qingyi Feng[3, §], Yeqing Yang[1], Xiaocong Shen[1], Kaizhi Chen[1], Long Yao,[1] Kunjie Zhu[1], Xiang-Long Huang[1,*], Zhe Hu[2,*], Hua-Kun Liu[1], Yun-Xiao Wang[1,*]

[1] Institute of Energy Materials Science, University of Shanghai for Science and technology, Shanghai 290000, P.R. China

E-mail: xlhuang_uestc@163.com; yunxiaowang@usst.edu.cn

[2] Guangdong Provincial Key Laboratory of New Energy Materials Service Safety, College of Materials Science and Engineering, Shenzhen University, Shenzhen 518060, P.R. China

E-mail: huzhe@szu.edu.cn;

[3] ShenSi Lab, Shenzhen Institute for Advanced Study, University of Electronic Science and Technology of China, Shenzhen 518110, P.R. China

[§] These authors equally contribute to this work.

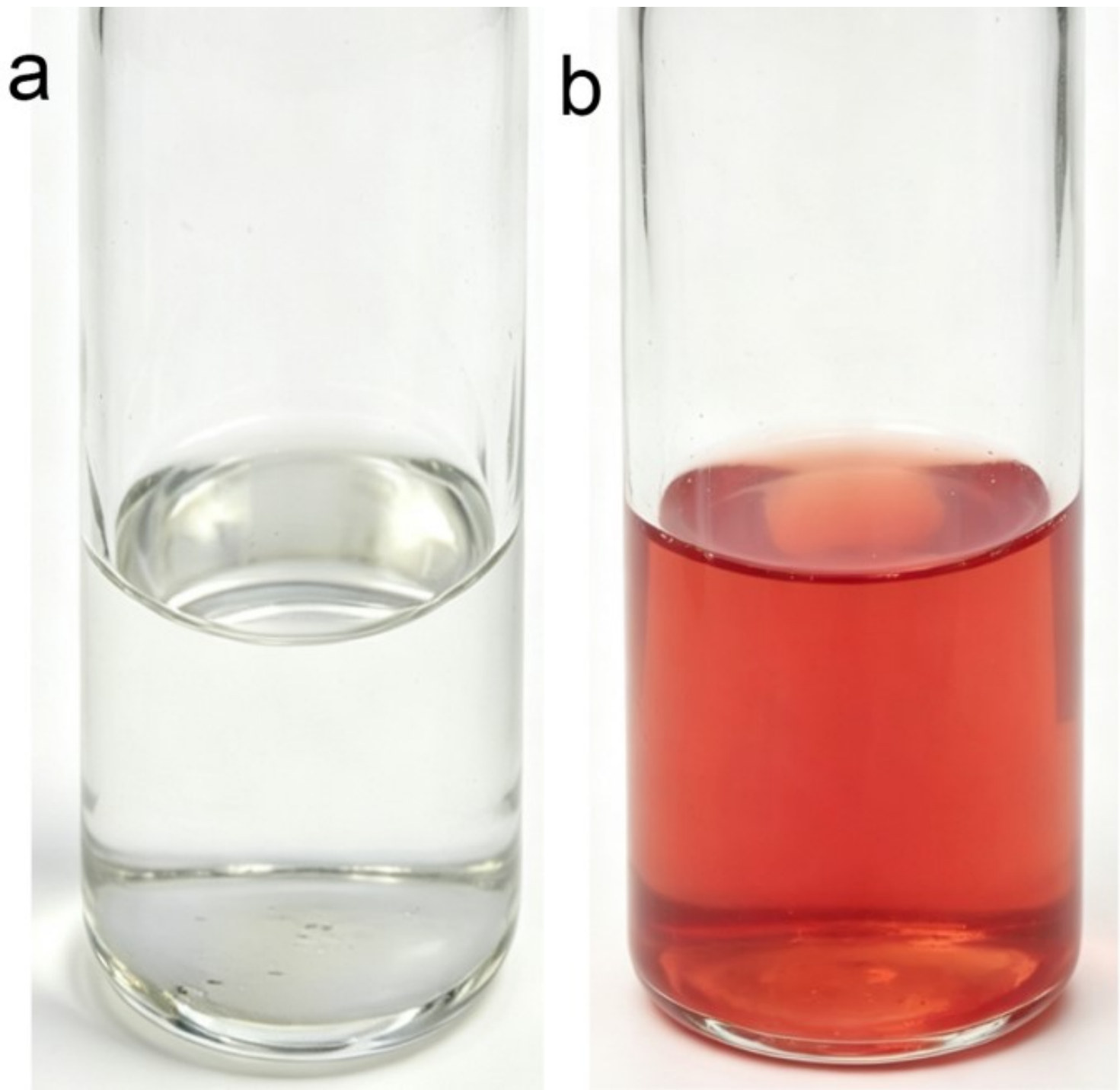

Figure S1. Photos of LHCE(a) and LHCE-SbI_3(b) electrolytes.

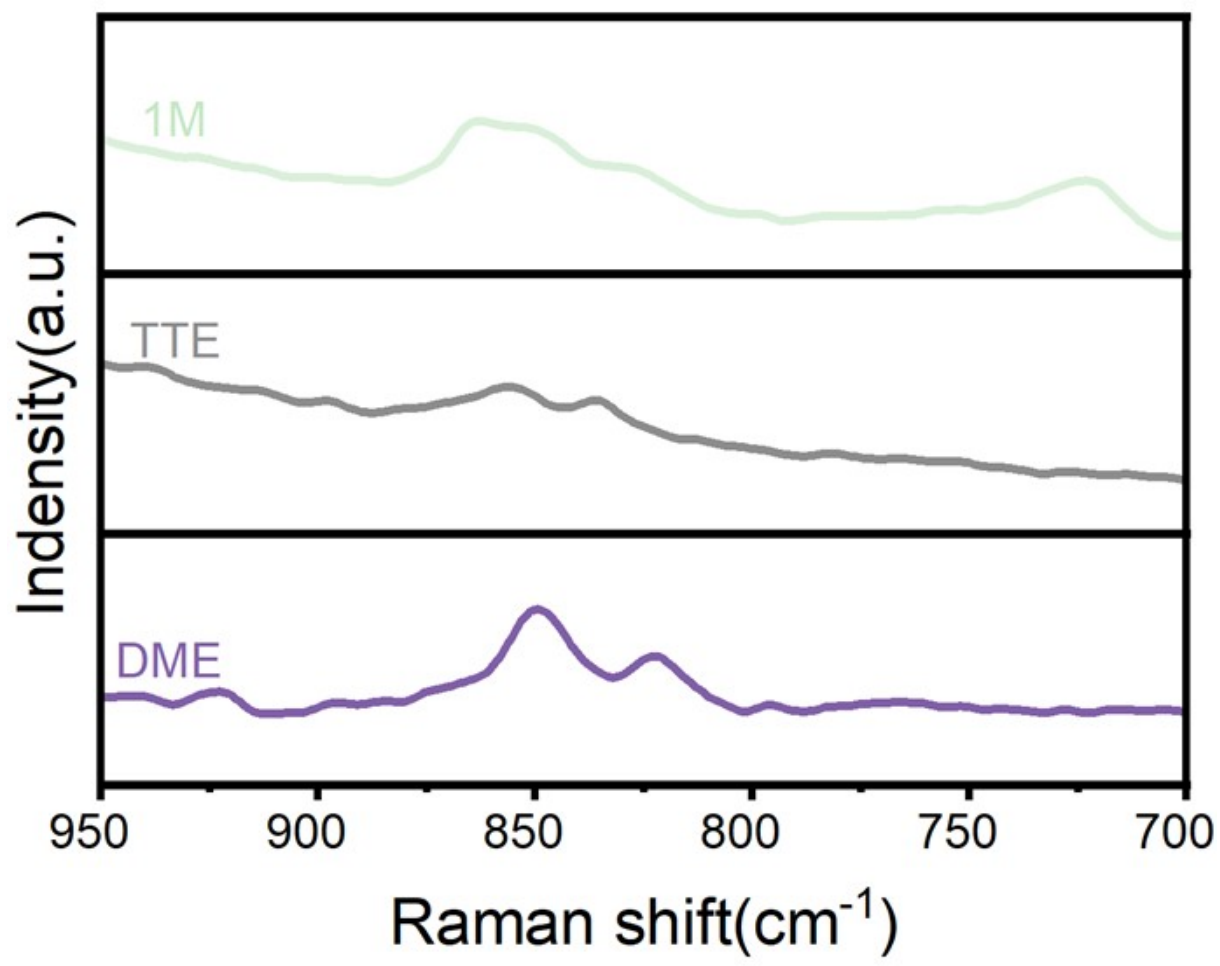

Figure S2. Raman spectra deconvolution of 1M,TTE and DME electrolytes.

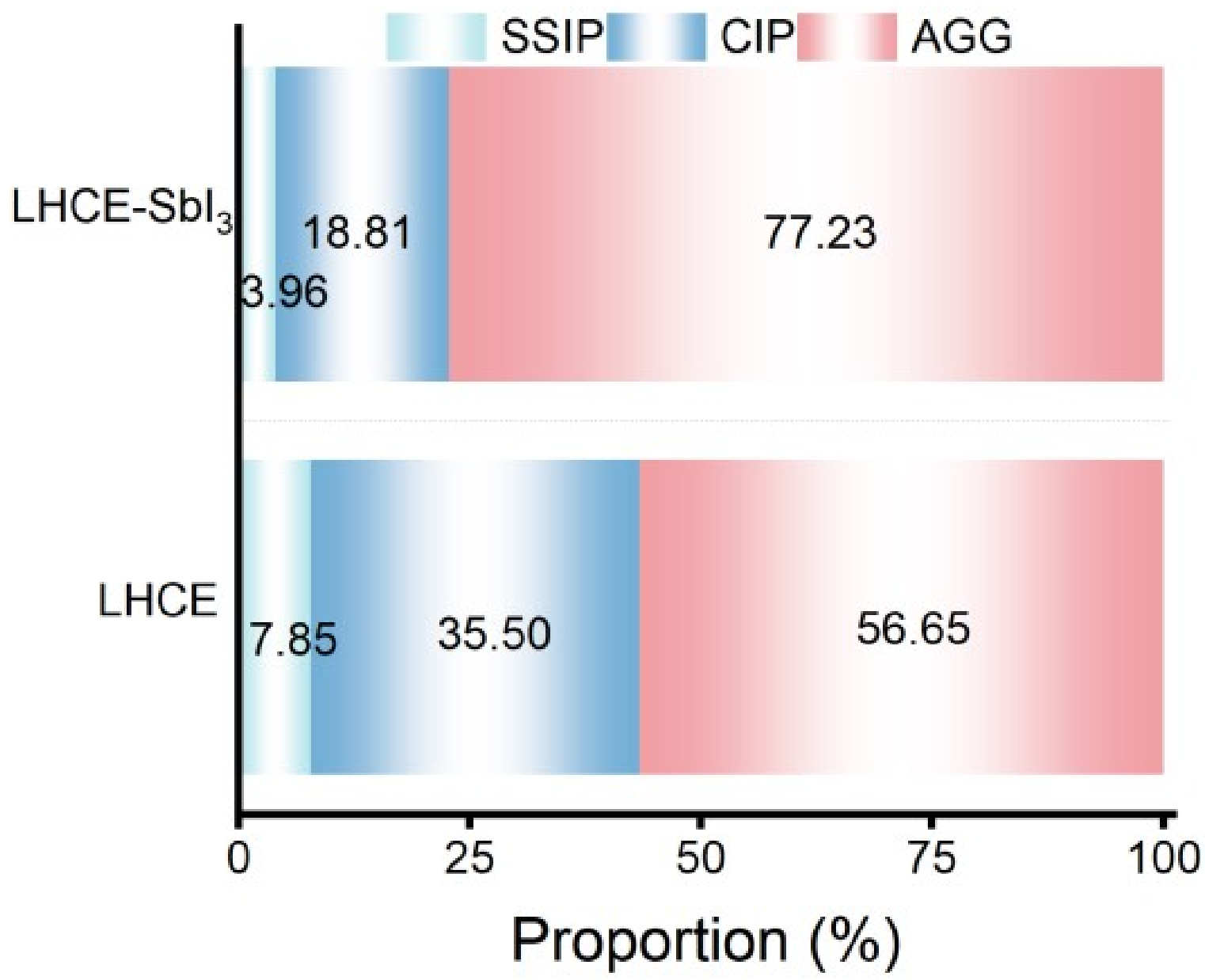

Figure S3. Calculation of the ratio of different coordination structures in various LHCEs derived from Raman spectra.

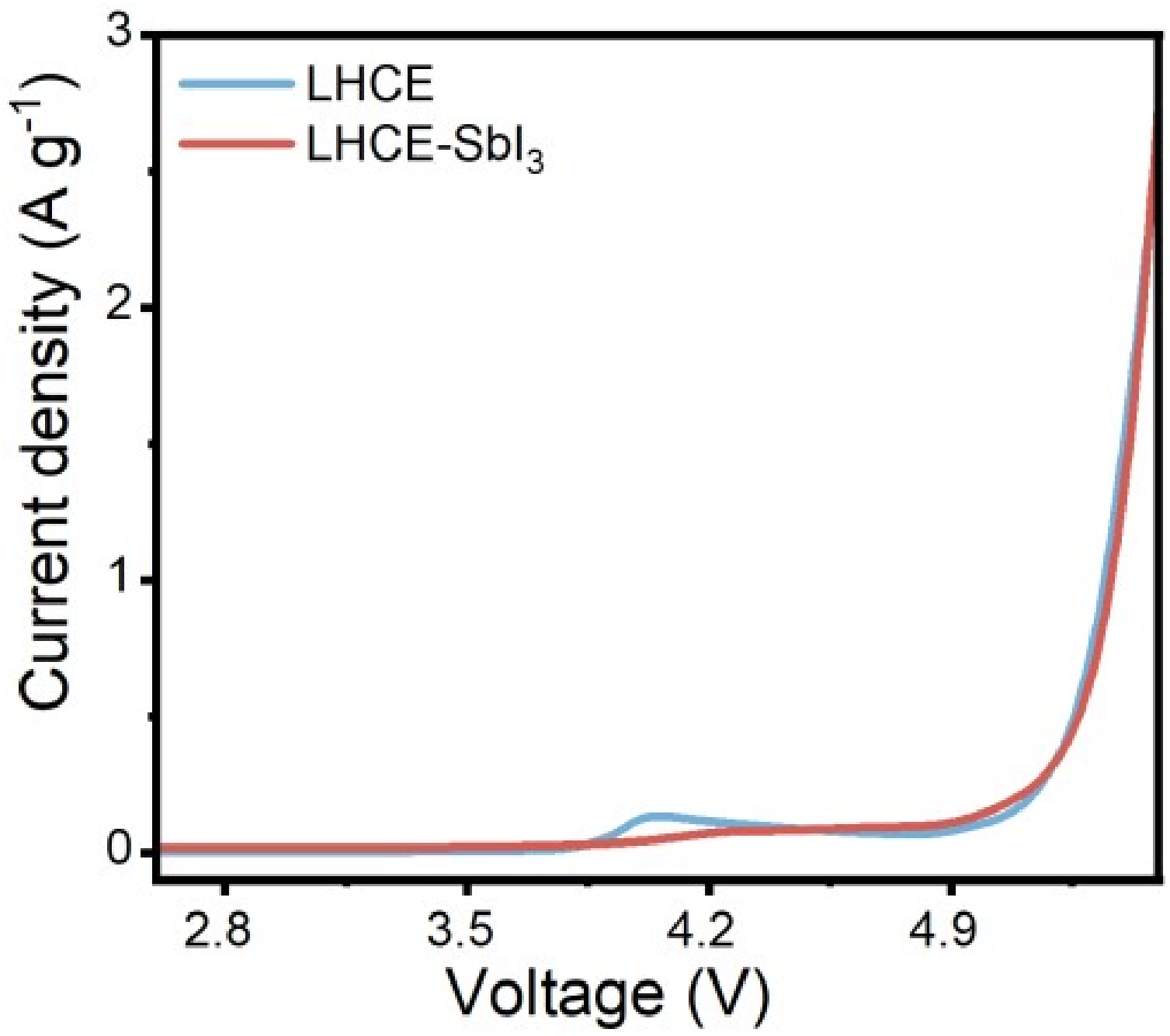

Figure S4. Linear sweep voltammetry of LHCE and LHCE-SbI_3 electrolytes.

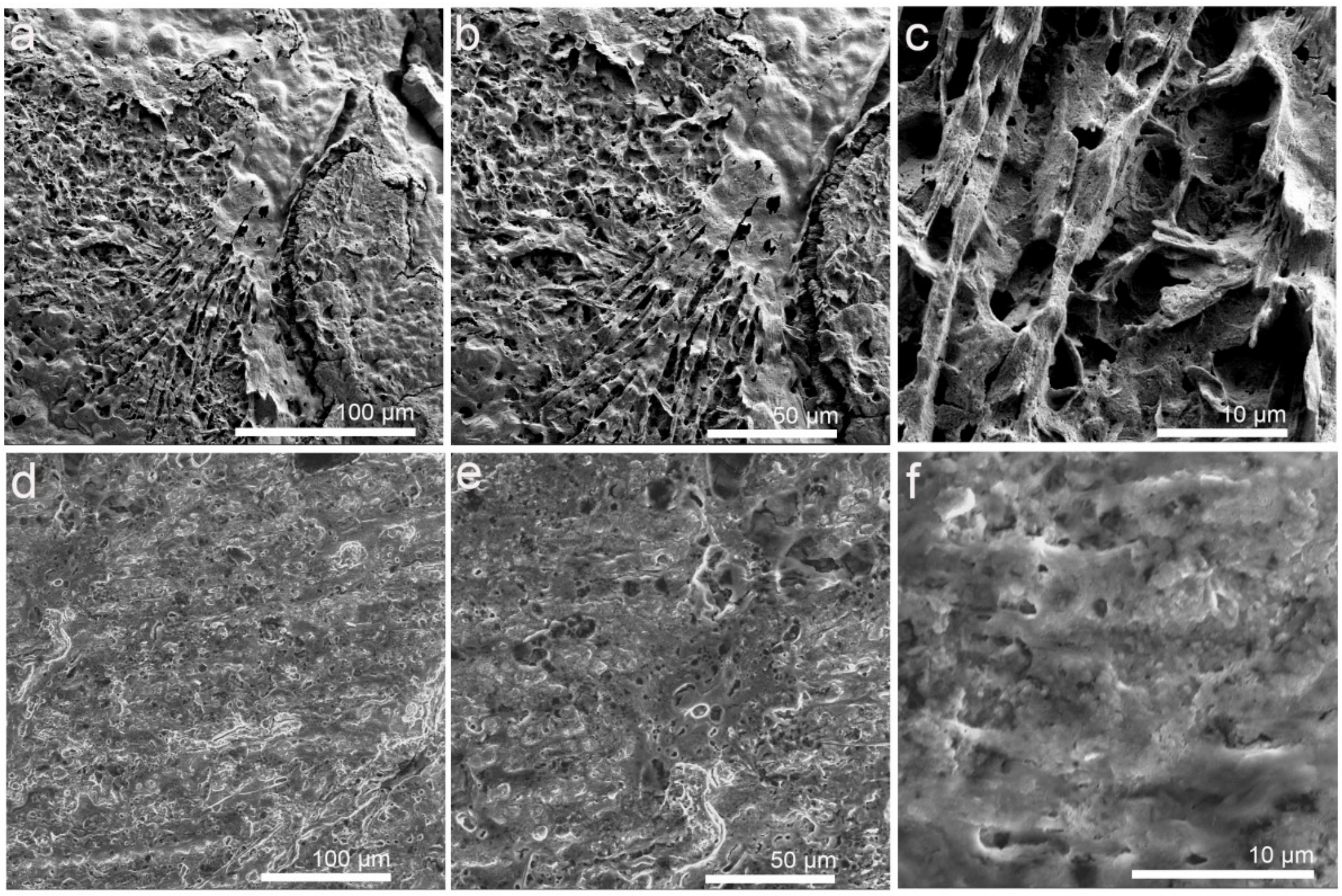

Figure S5.SEM images of sodium metal from the Na||Na symmetric cells after cycling for 20 h.

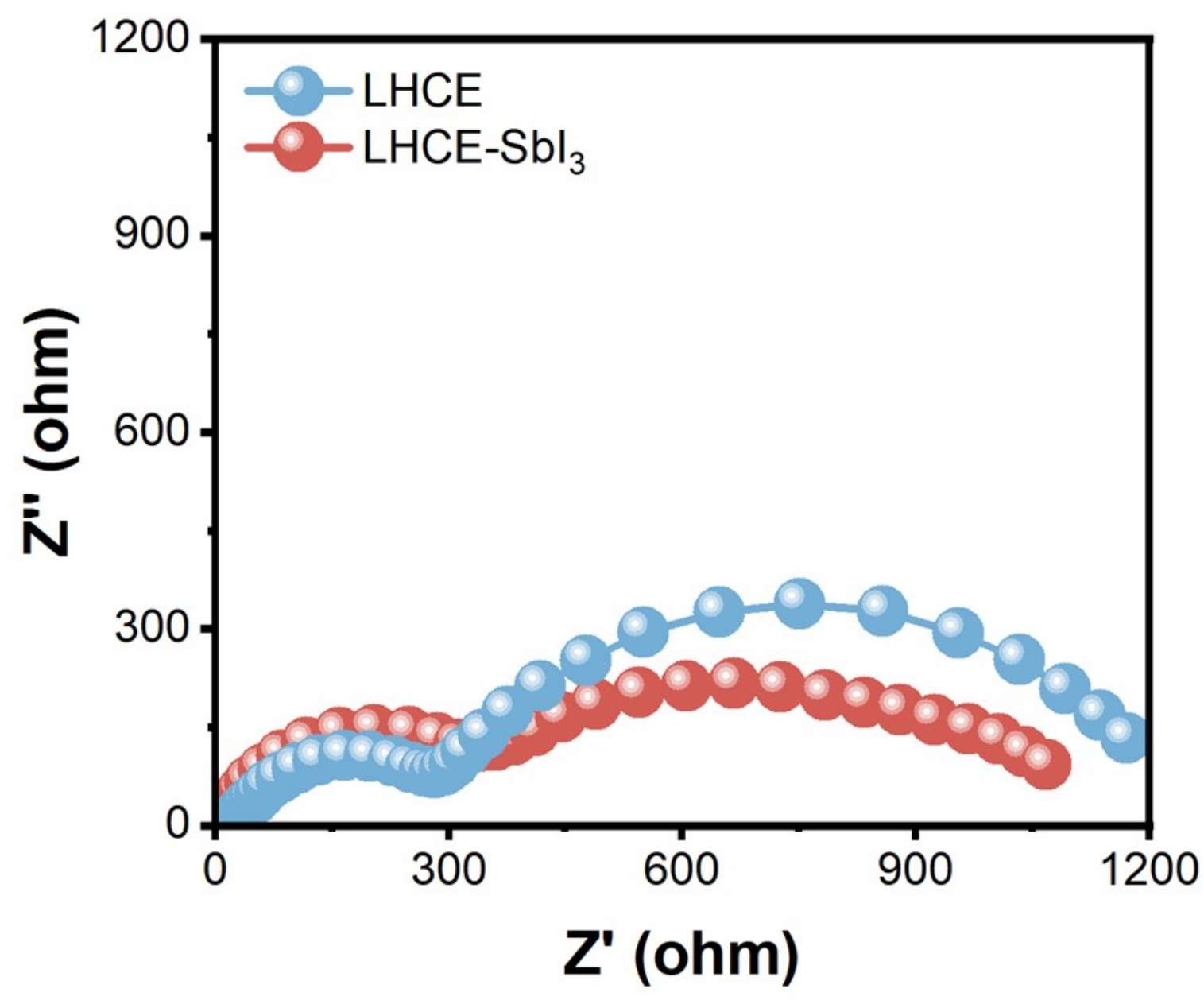

Figure S6.EIS curves for the LHCE and LHCE-SbI_3 on Na||Na symmetric cells.

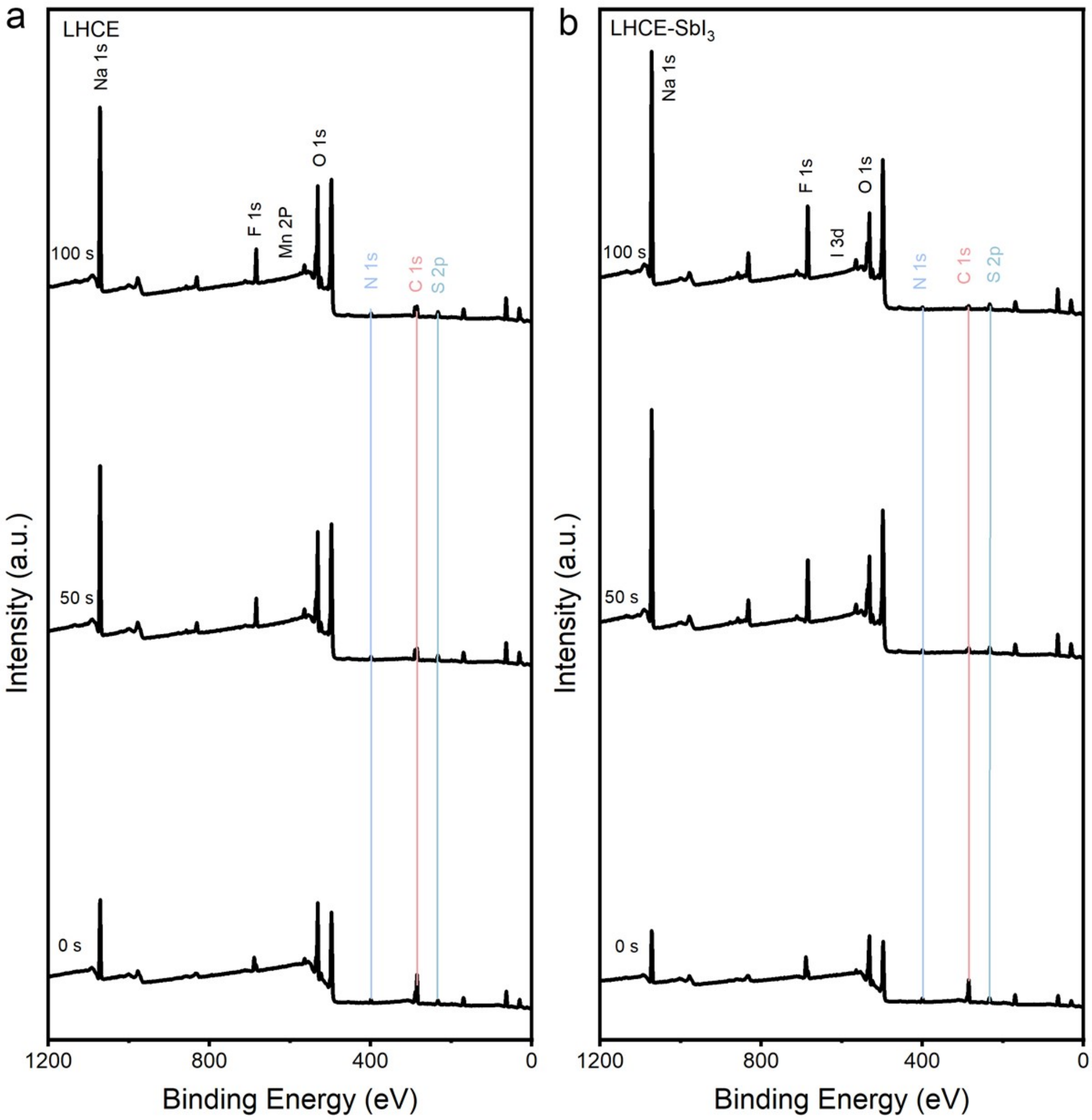

Figure S7.XPS survey spectra of the electrode surfaces cycled in LHCE(a) and LHCE-SbI_3(b) electrolytes at different sputtering times (0 s, 50 s, and 100 s).

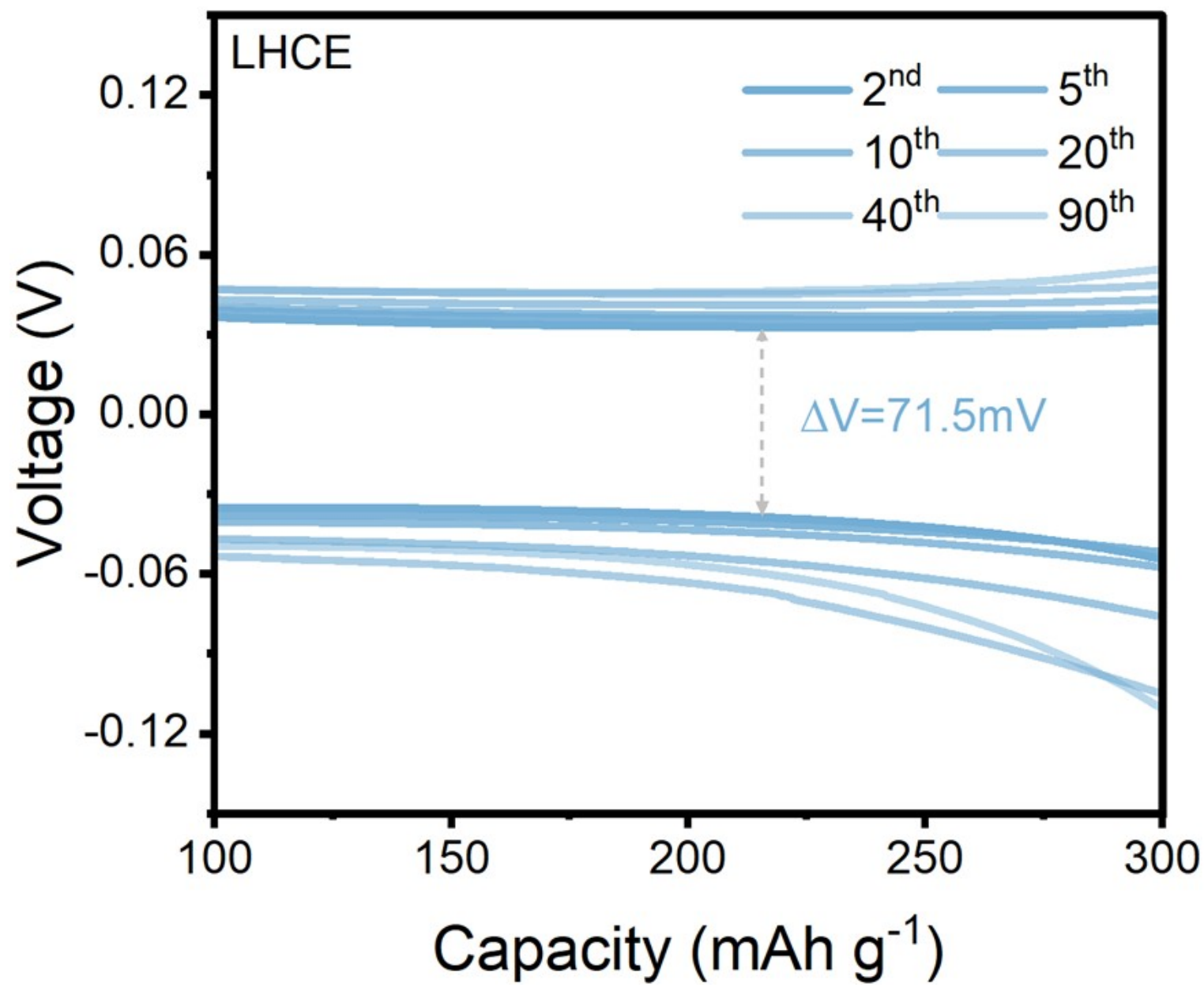

Figure S8. Voltage-capacity profiles of Na||Cu half cell with the LHCE electrolyte at selected cycles.

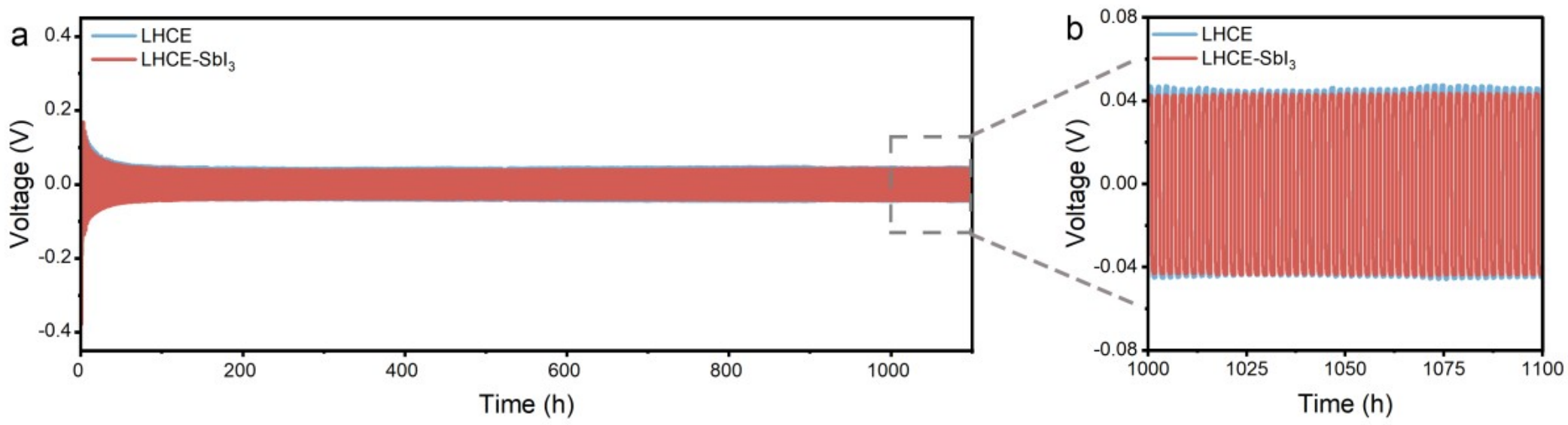

Figure S9. (a) Long-term cycling voltage-time profiles of Na||Na symmetric cells with the LHCE and LHCE-SbI_3 electrolytes at a current density of 0.5 mA cm^{-2}. (b) Enlarged view of the region spanning 1000 to 1100 hours.

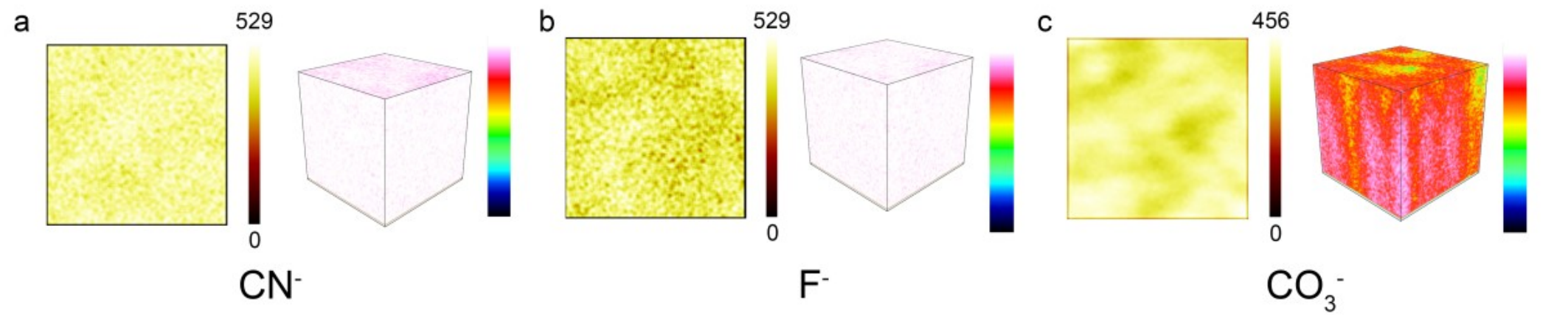

Figure S10. 2D and 3D reconstructed TOF-SIMS mappings of characteristic ion fragments for the SPAN cathode after cycling.

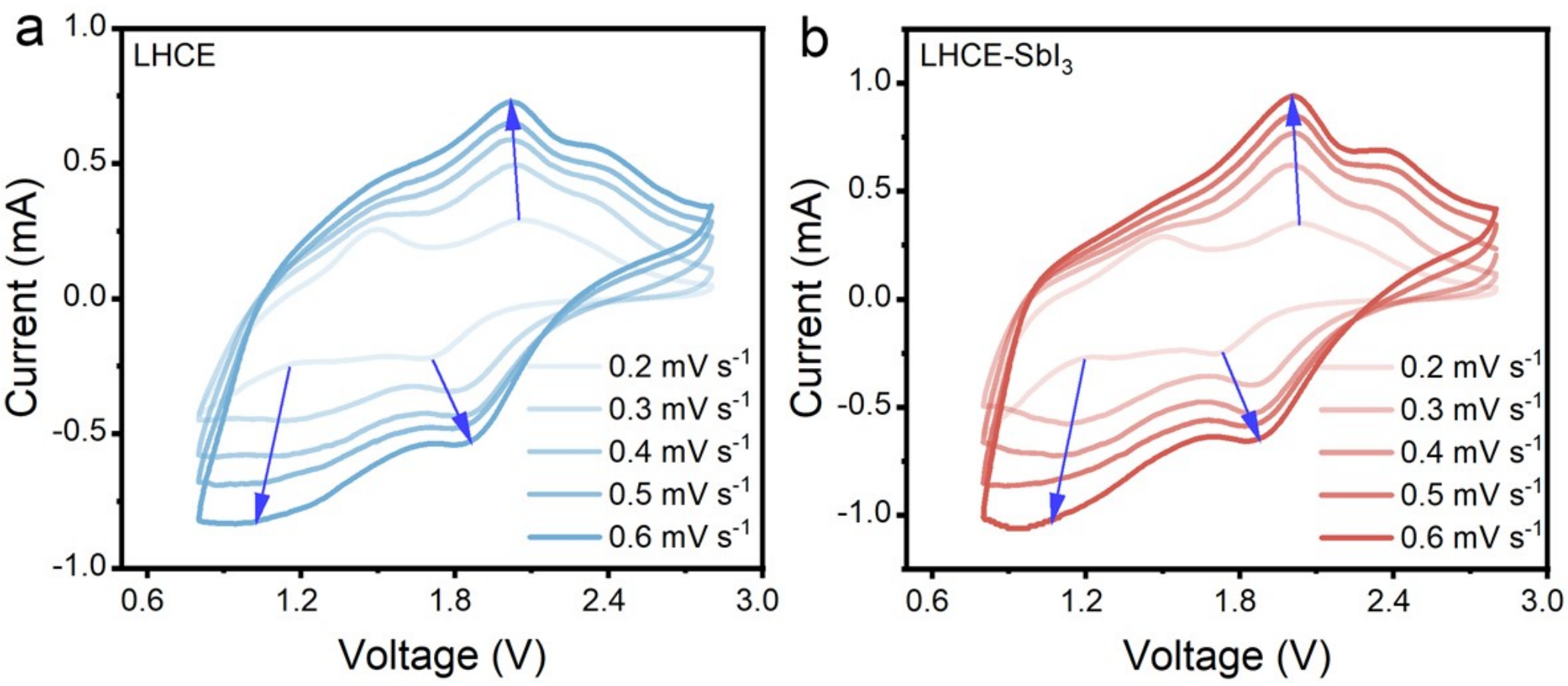

Figure S11.CV profiles of the Na-S cells with different LHCEs at various scan rates.

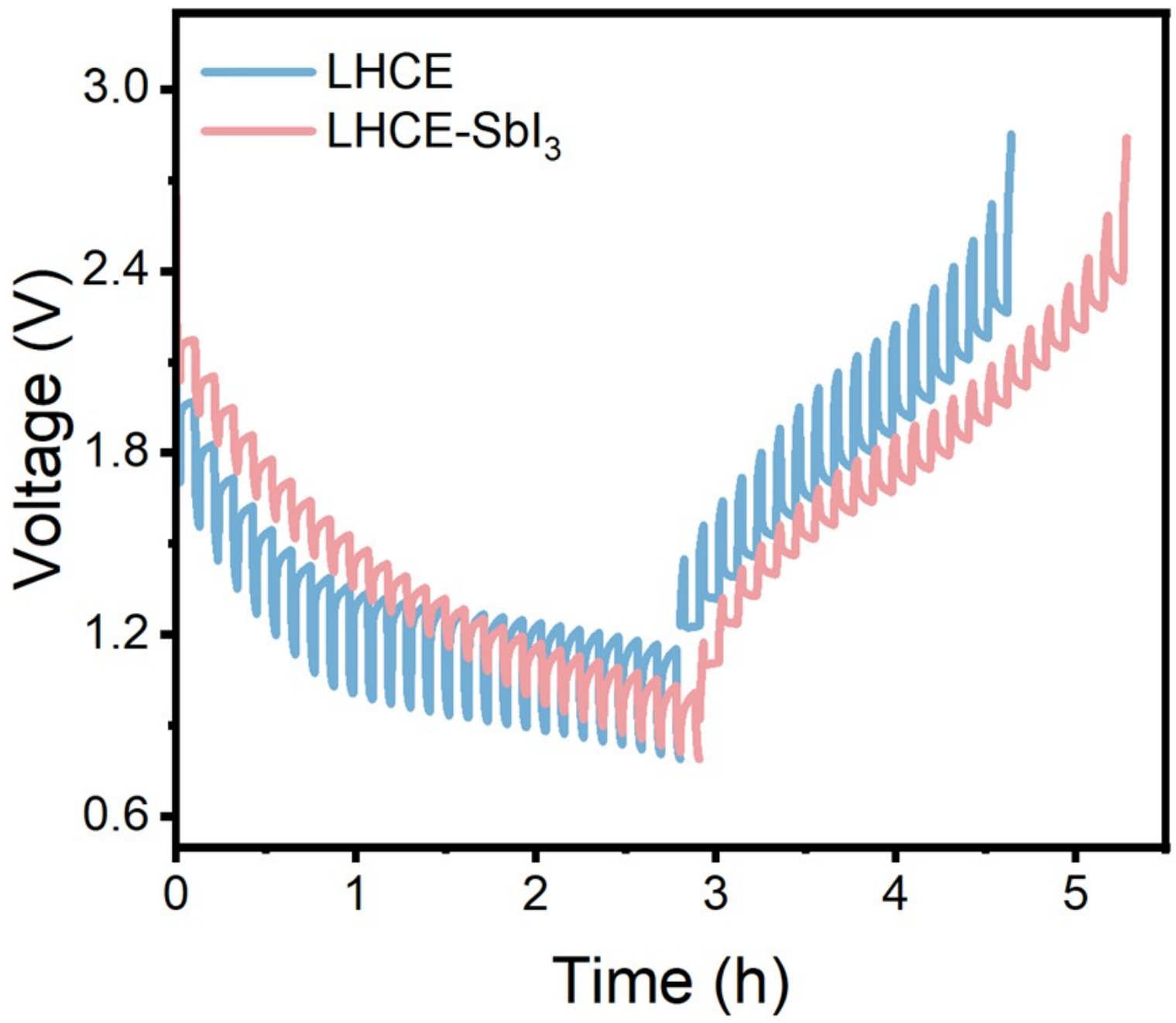

Figure S12.GITT voltage profiles of the Na-S cells with different LHCEs as a function of time.

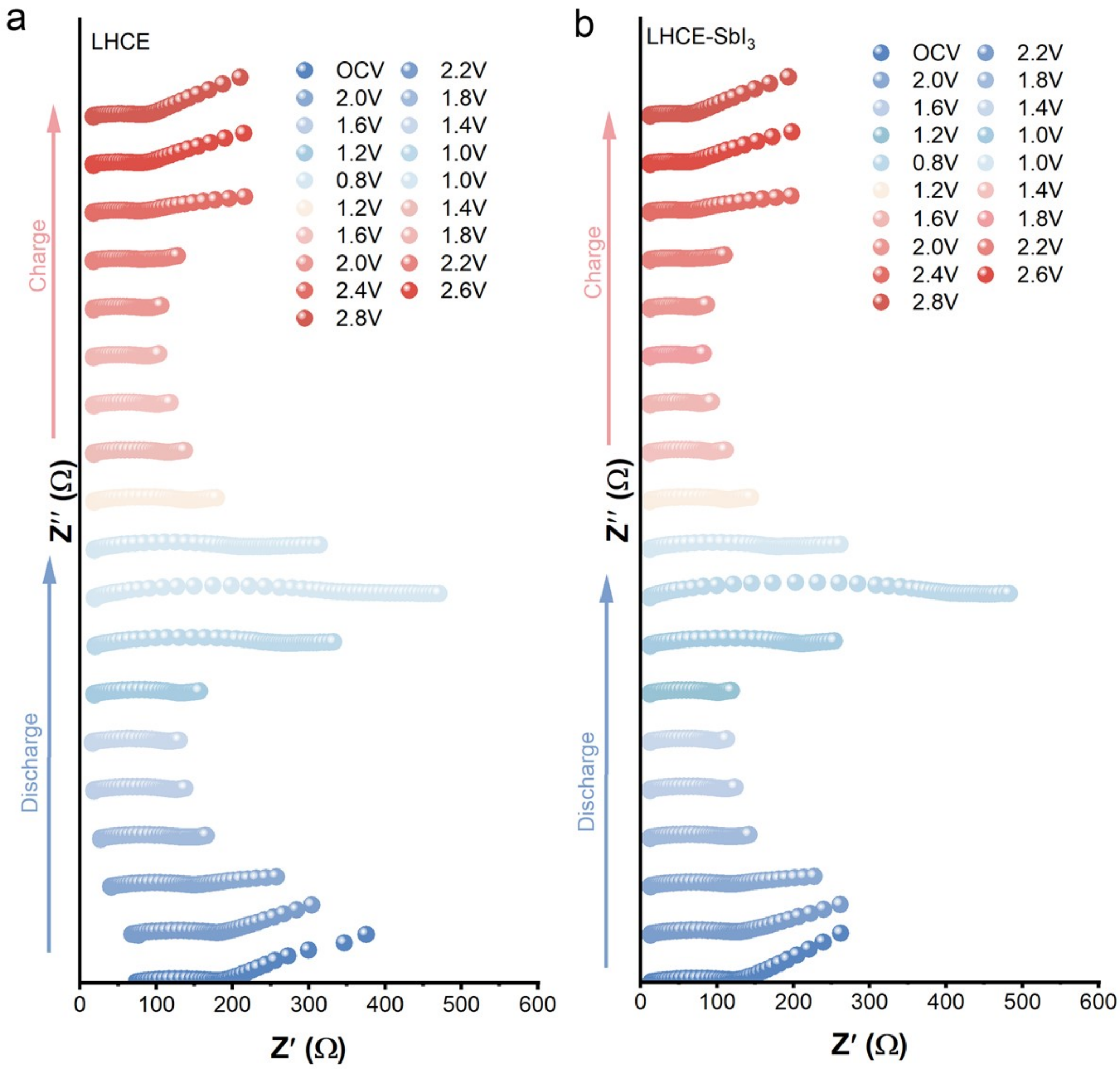

Figure S13. In-situ EIS curves for the LHCE and LHCE-SbI_3 Na-S cells.

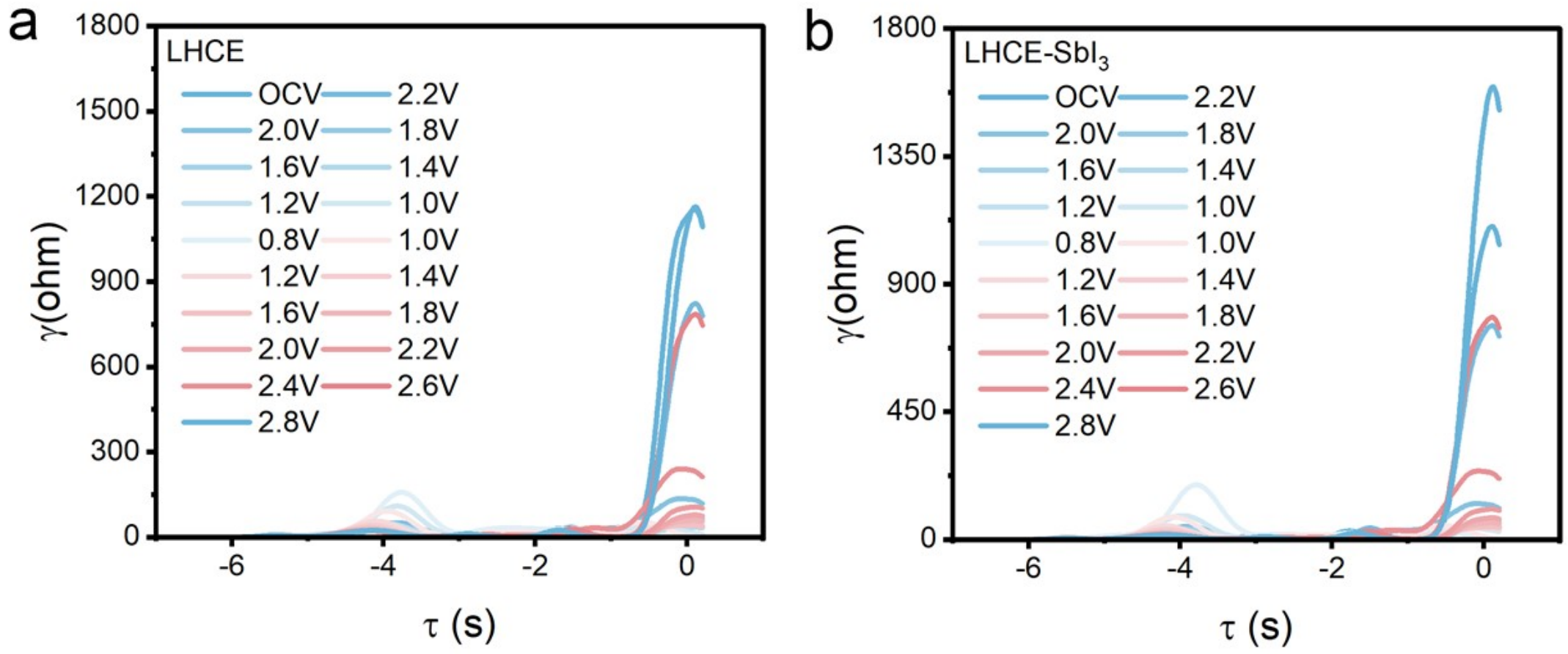

Figure S14. Decoupling of DRT peak derived from the in-situ EIS curves.

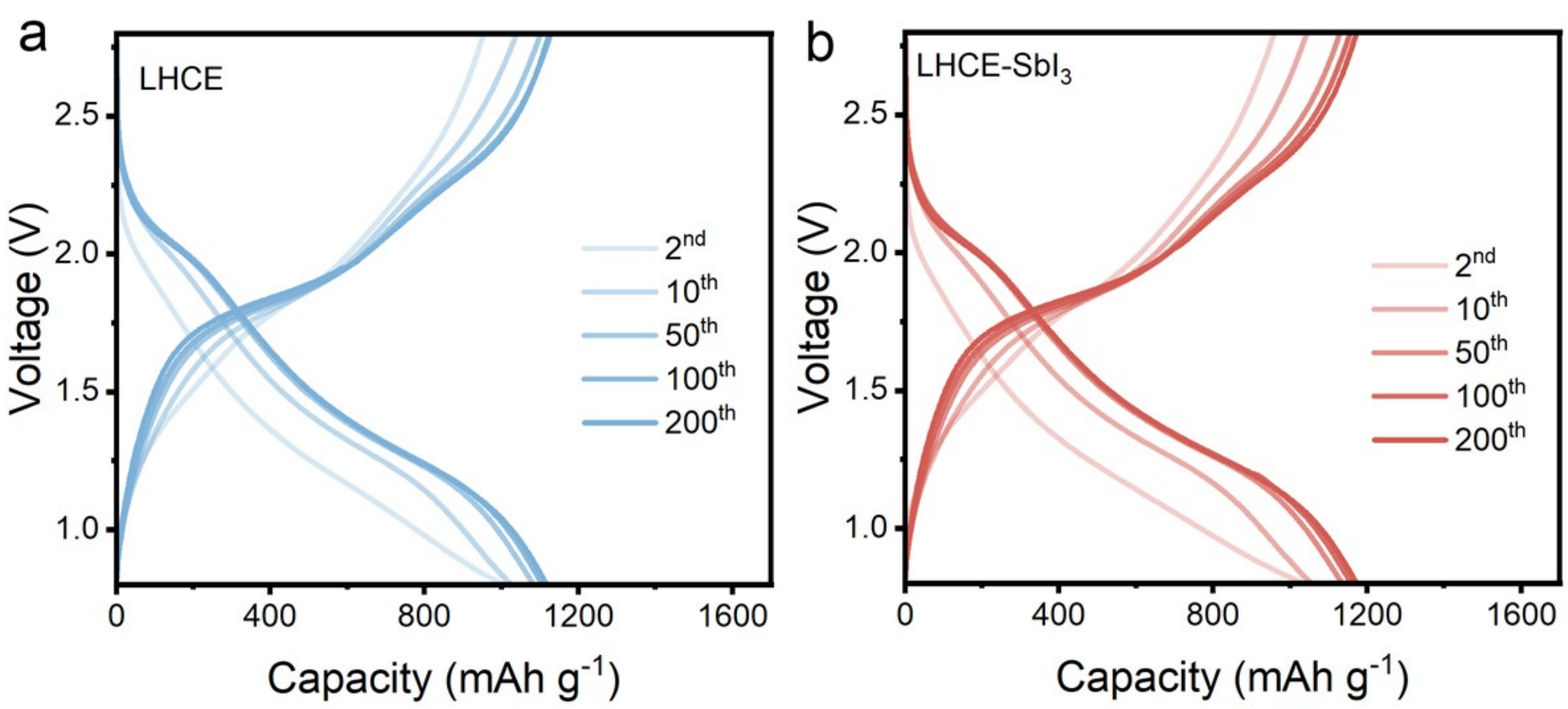

Figure S15. The discharge-charge profiles at the selected cycles at 0.2 C.

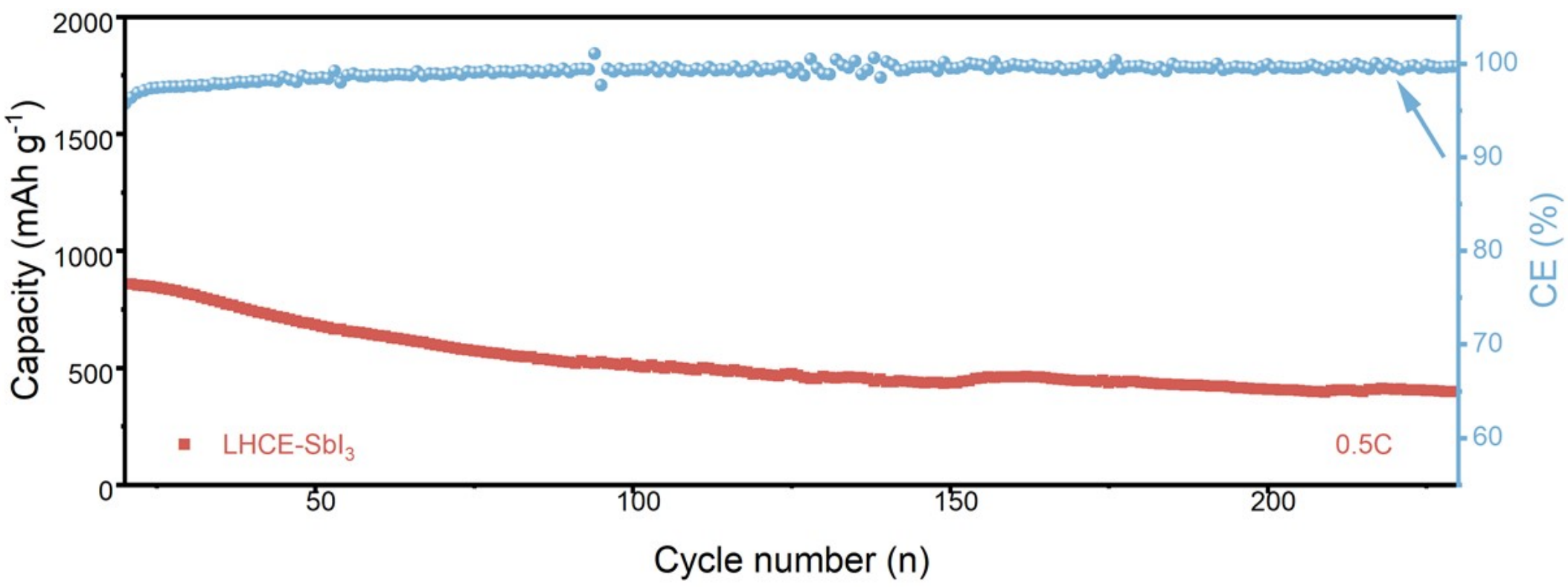

Figure S16. Cycling performance of the LHCE-SbI_3 cell with a high areal loading of 2mg cm^{-2} at 0.5 C.

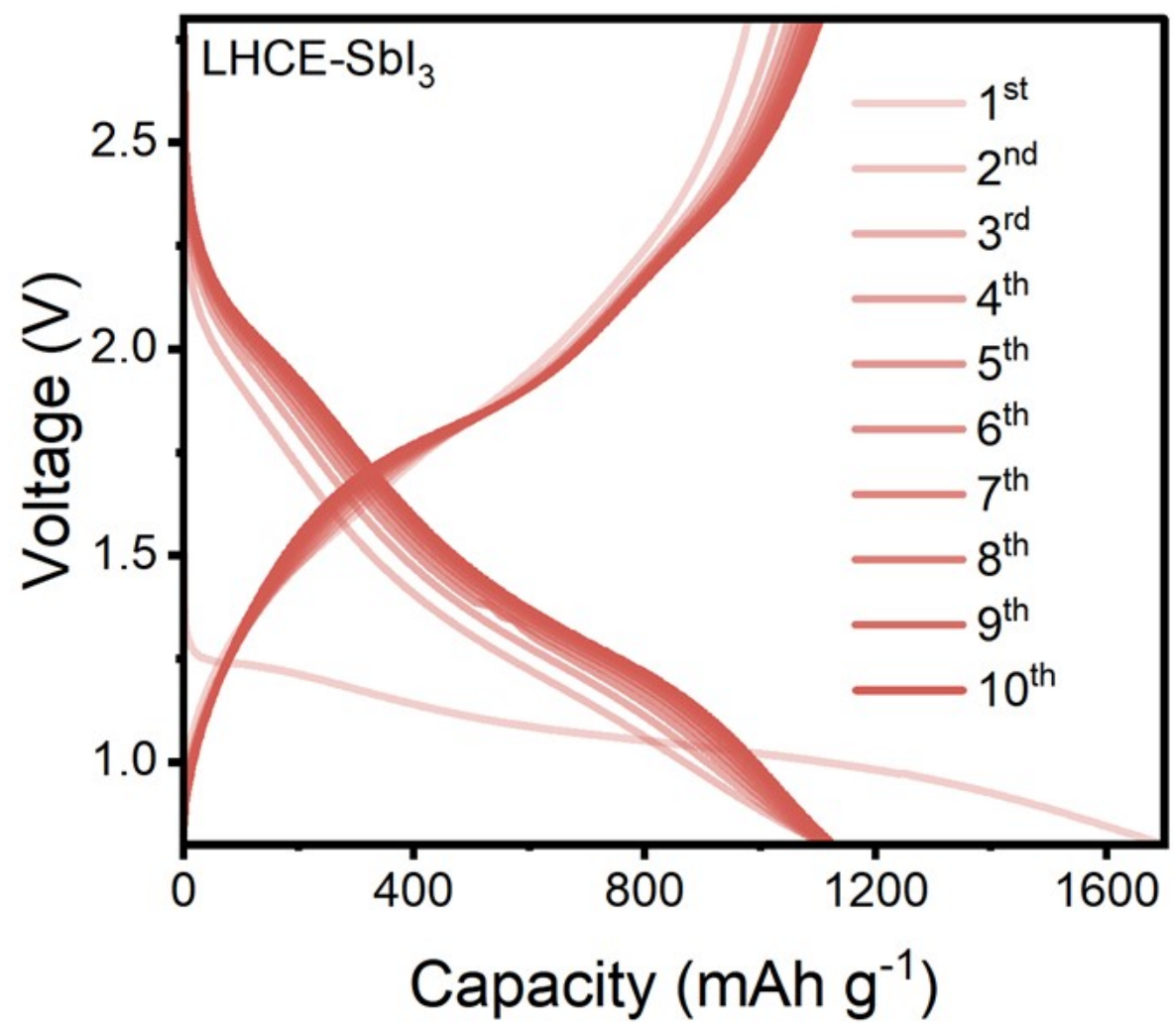

Figure S17. Charge-discharge profiles of the initial 10 cycles at 0.2 C prior to long-term cycling at 1.0 C.
