# Supplementary material for: In situ iodine-grafted SPAN via a decoupled additive for durable and high-rate Na–S batteries
Source: Chem Sci. 2026 Sep 18. Online ahead of print. doi: 10.1039/d6sc06580e (PMC13588261; doi:10.1039/d6sc06580e)
Supplement: Supplementary file 1 [file SC-OLF-D6SC06580E-s001.pdf]

Supplementary Information (SI) for Chemical Science.
This journal is © The Royal Society of Chemistry 2026

# Supporting Information

## In-Situ Iodine-Grafted SPAN via a Bifunctional Additive for Durable and High-Rate Na-S Batteries

Mingyue Yang[§,1], Xue Li[2§], Qingyi Feng[3, §], Yeqing Yang[1], Xiaocong Shen[1], Kaizhi Chen[1], Long Yao,[1] Kunjie Zhu[1], Xiang-Long Huang[1,*], Zhe Hu[2,*], Hua-Kun Liu[1], Yun-Xiao Wang[1,*]

[1] Institute of Energy Materials Science, University of Shanghai for Science and technology, Shanghai 290000, P.R. China

E-mail: xlhuang_uestc@163.com; yunxiaowang@usst.edu.cn

[2] Guangdong Provincial Key Laboratory of New Energy Materials Service Safety, College of Materials Science and Engineering, Shenzhen University, Shenzhen 518060, P.R. China

E-mail: huzhe@szu.edu.cn;

[3] ShenSi Lab, Shenzhen Institute for Advanced Study, University of Electronic Science and Technology of China, Shenzhen 518110, P.R. China

[§] These authors equally contribute to this work.

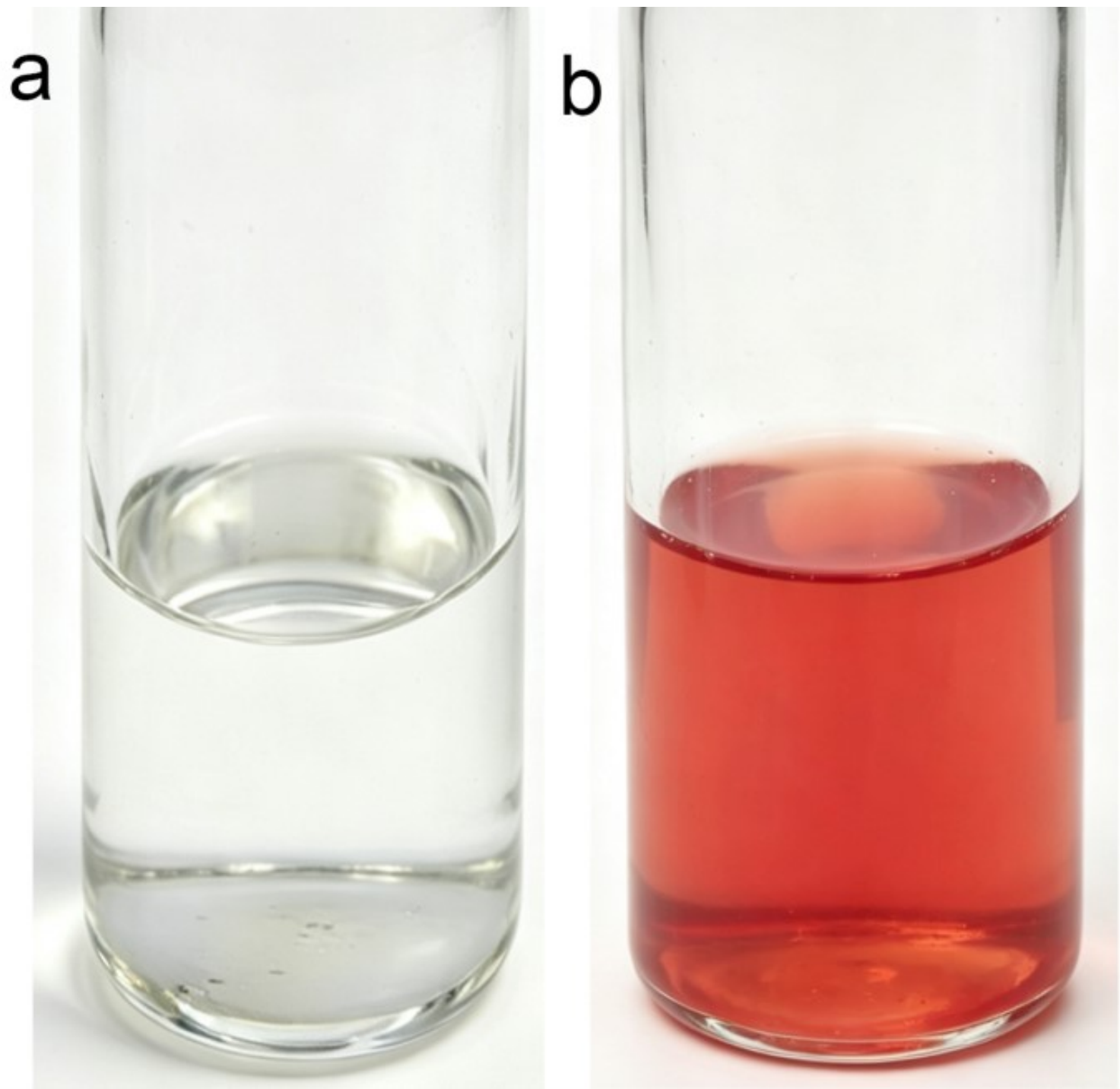


**Figure S1.**Photos of LHCE(a) and LHCE-$SbI_3$(b) electrolytes.

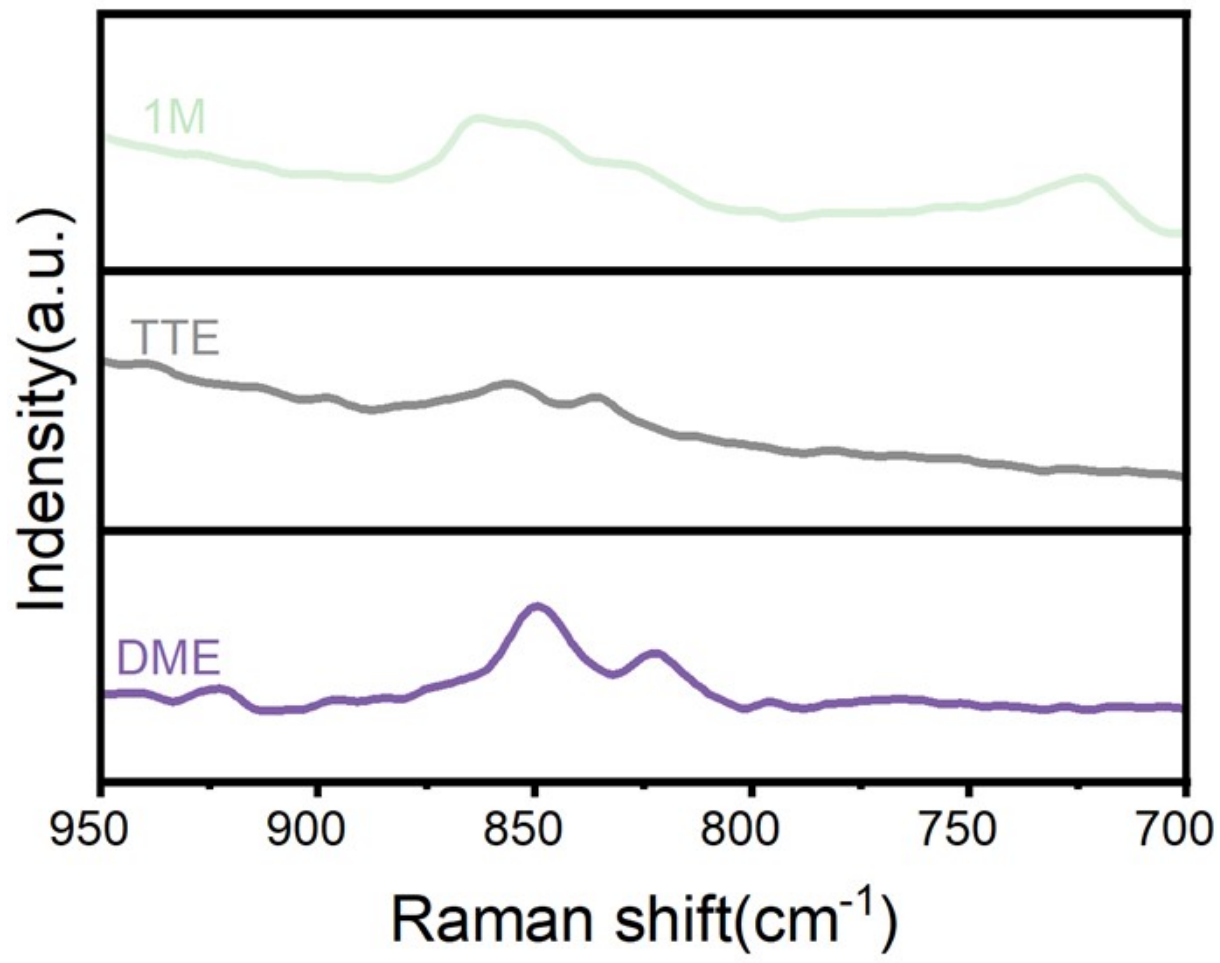


**Figure S2.** Raman spectra deconvolution of 1M,TTE and DME electrolytes.

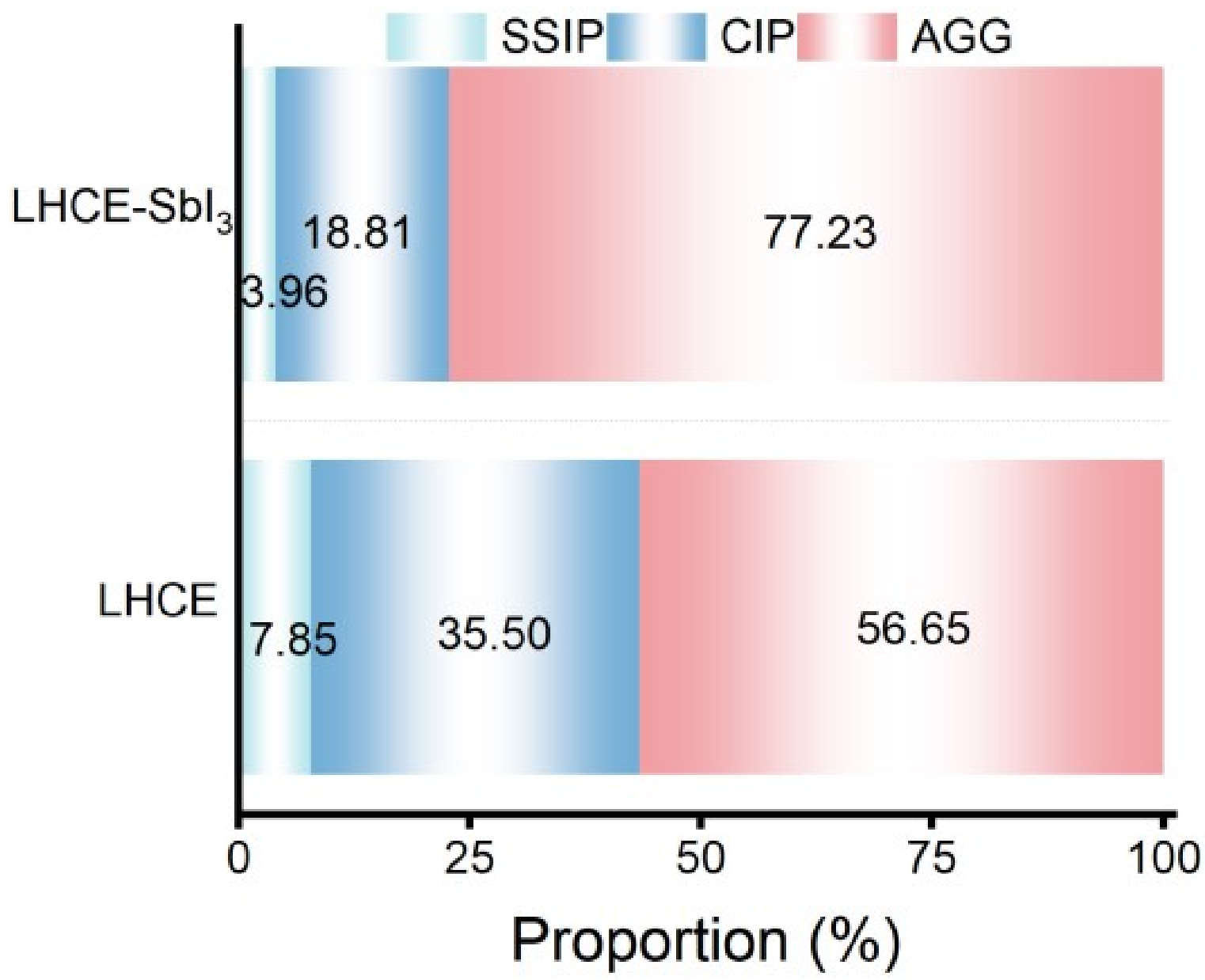


**Figure S3.**Calculation of the ratio of different coordination structures in various LHCEs derived from Raman spectra.

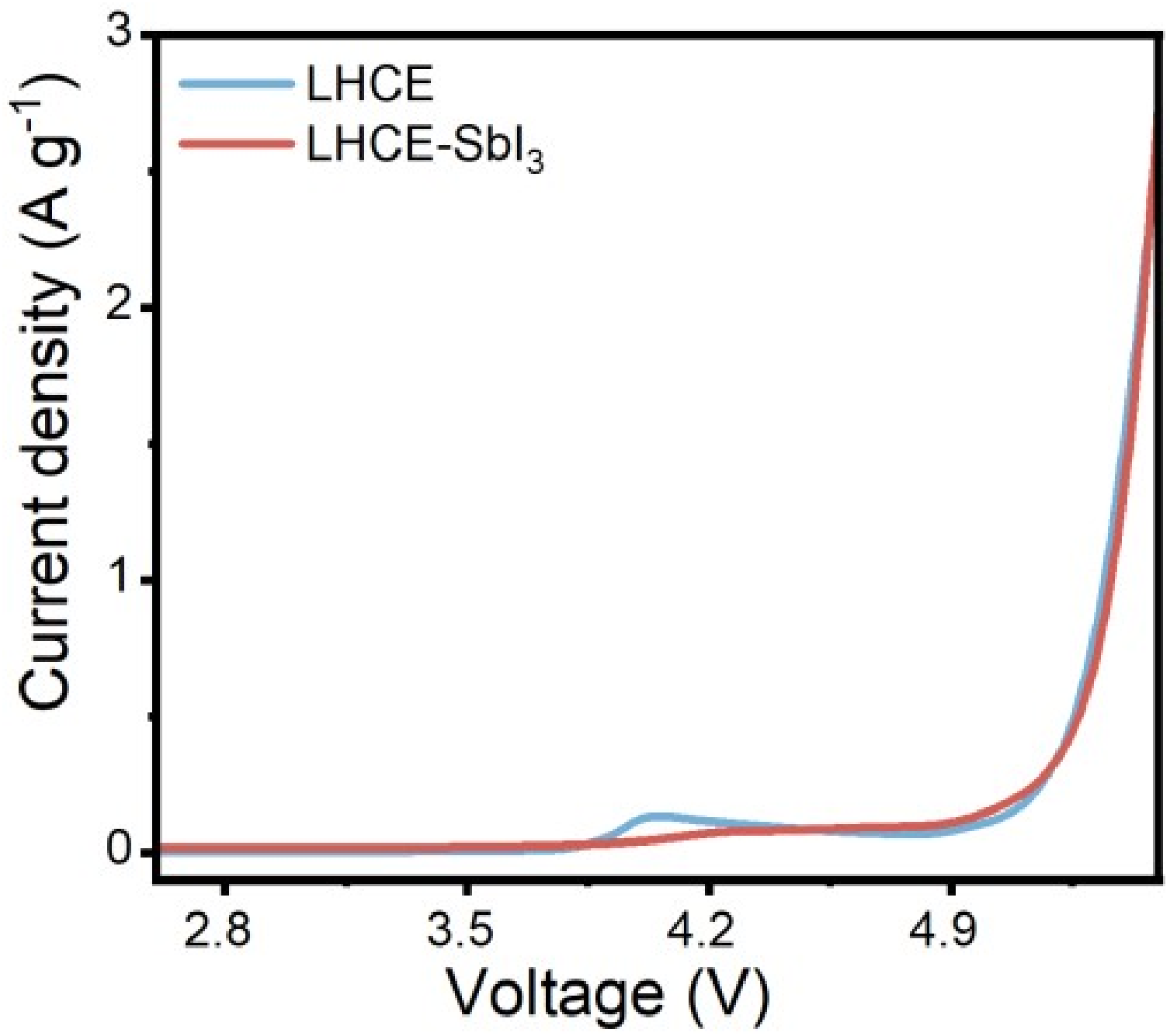


**Figure S4.**Linear sweep voltammetry of LHCE and LHCE-$SbI_3$ electrolytes.

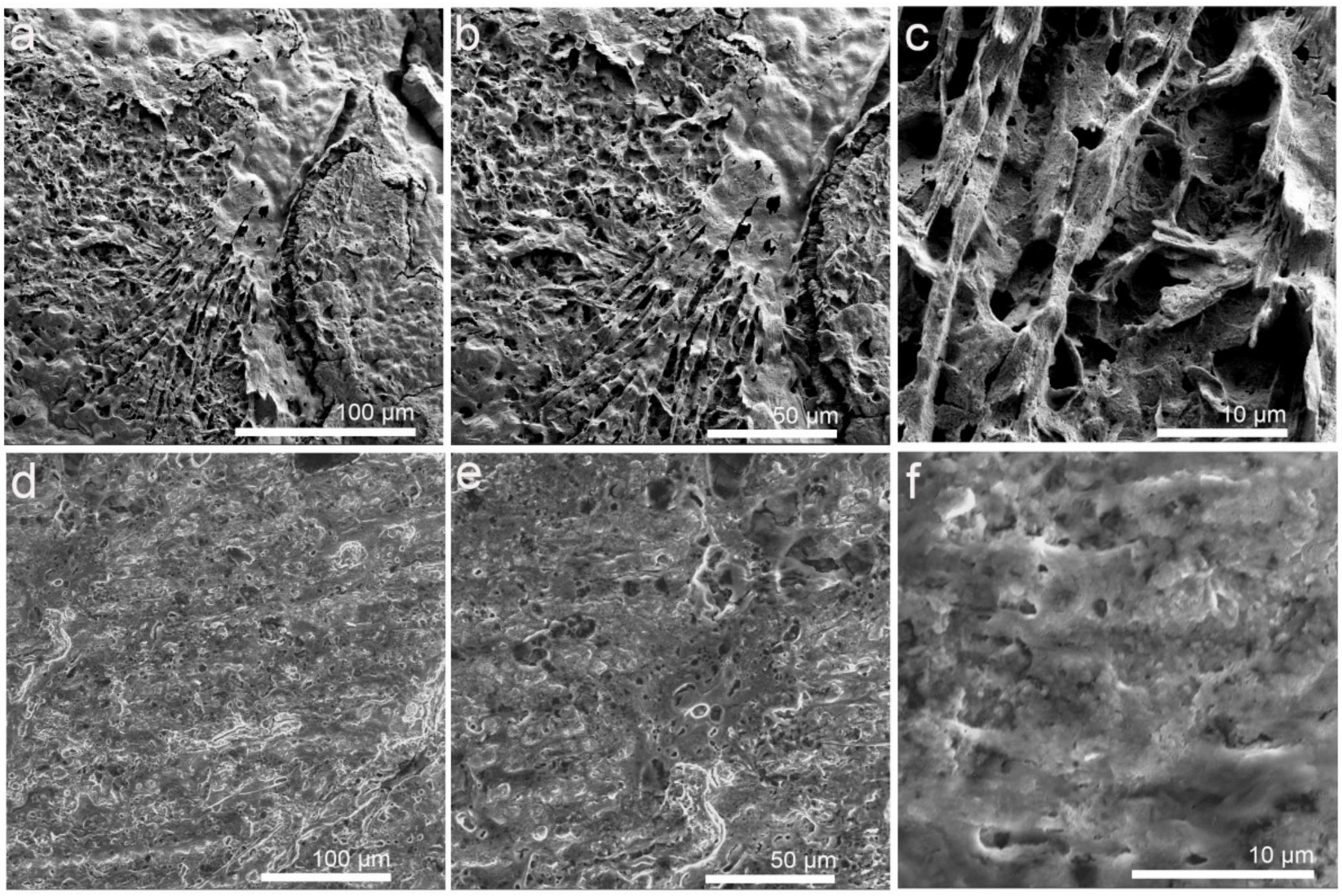


**Figure S5.**SEM images of sodium metal from the Na||Na symmetric cells after cycling for 20 h.

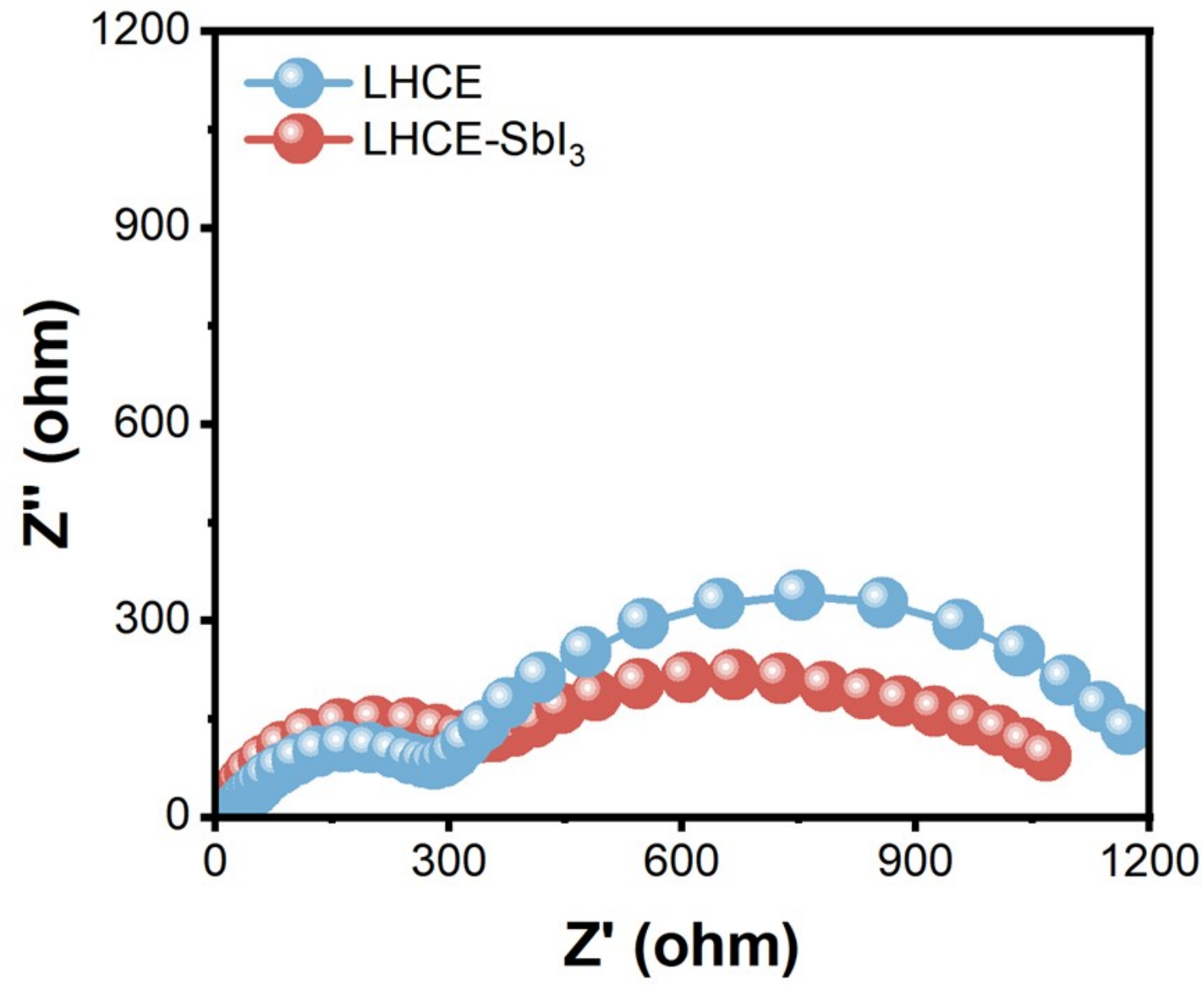


**Figure S6.**EIS curves for the LHCE and LHCE-$SbI_3$ on Na||Na symmetric cells.

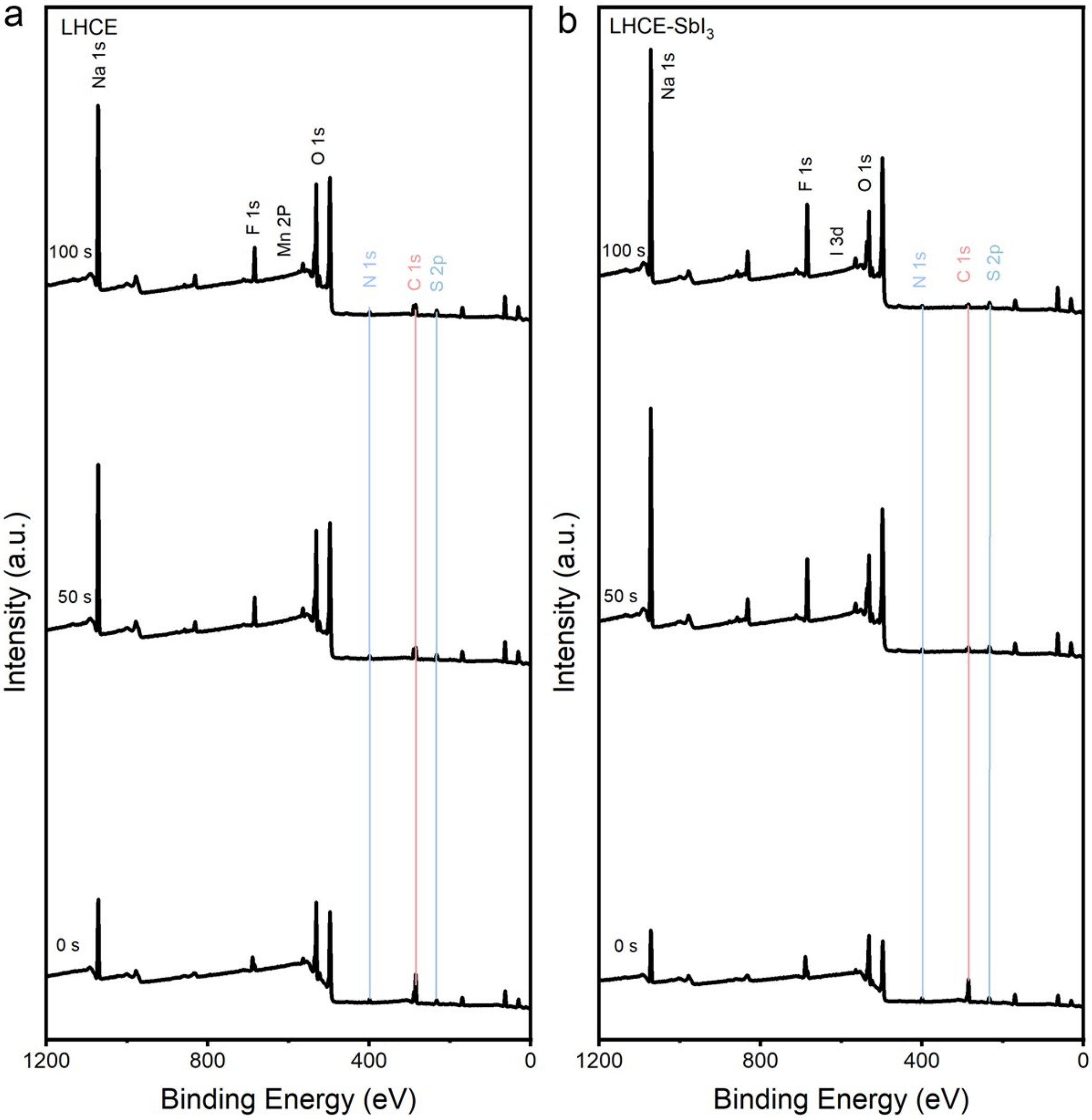


**Figure S7.**XPS survey spectra of the electrode surfaces cycled in LHCE(a) and LHCE-$SbI_3$(b) electrolytes at different sputtering times (0 s, 50 s, and 100 s).

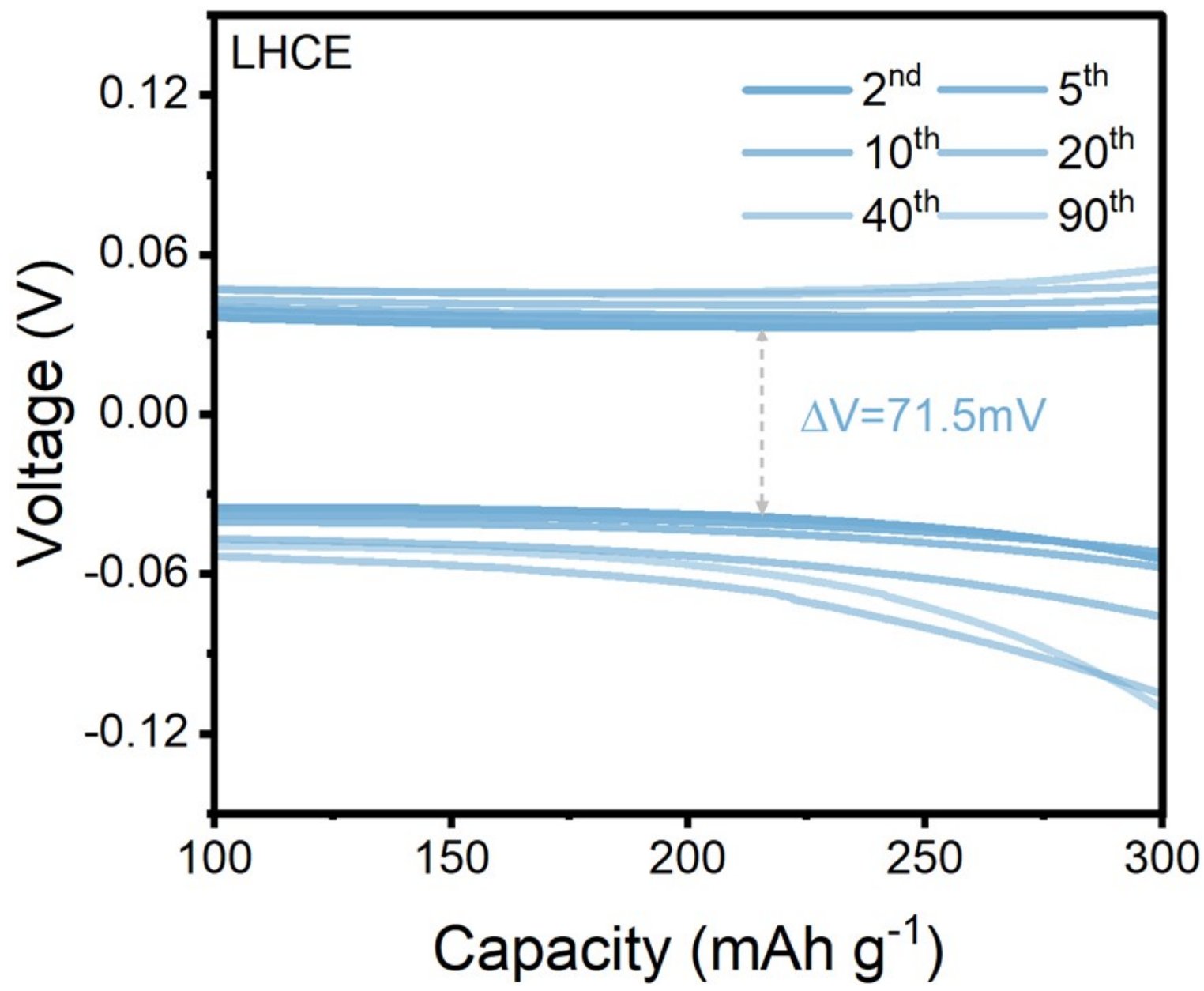


**Figure S8.** Voltage-capacity profiles of Na||Cu half cell with the LHCE electrolyte at selected cycles.

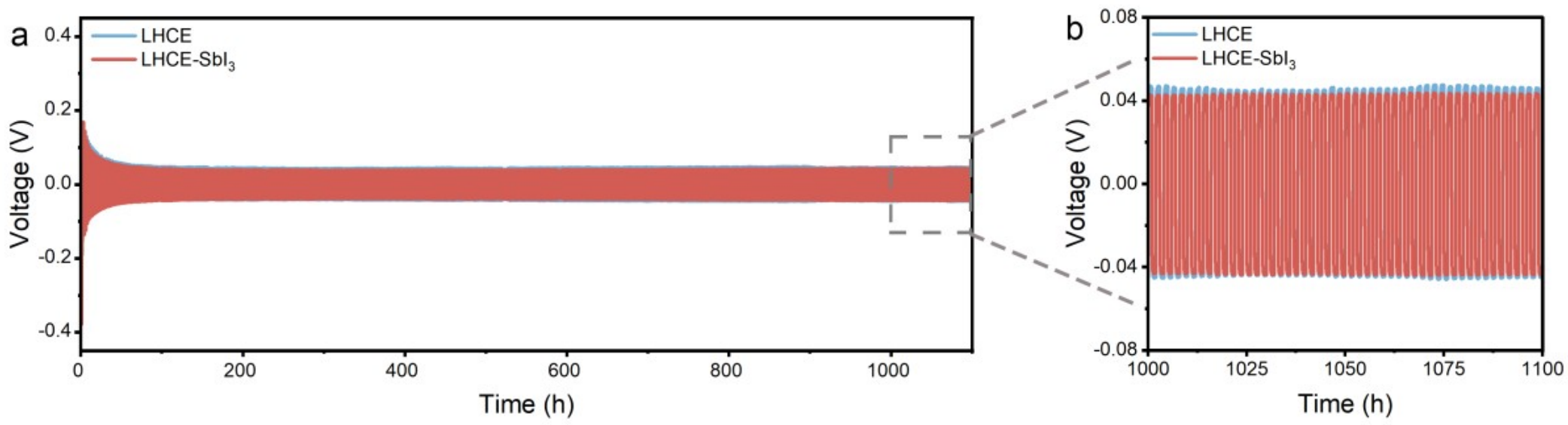


**Figure S9.** (a) Long-term cycling voltage-time profiles of Na||Na symmetric cells with the LHCE and LHCE-$SbI_3$ electrolytes at a current density of 0.5 mA $cm^{-2}$. (b) Enlarged view of the region spanning 1000 to 1100 hours.

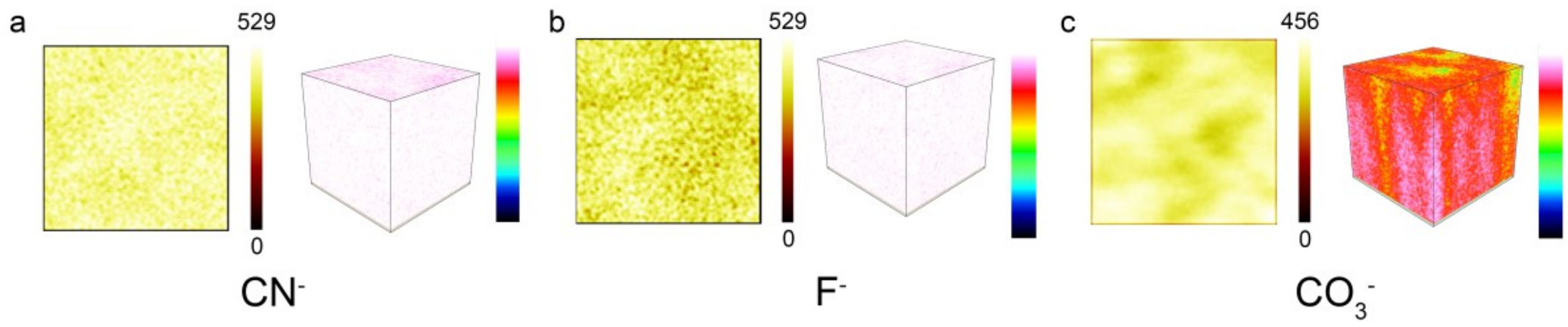


**Figure S10.** 2D and 3D reconstructed TOF-SIMS mappings of characteristic ion fragments for the SPAN cathode after cycling.

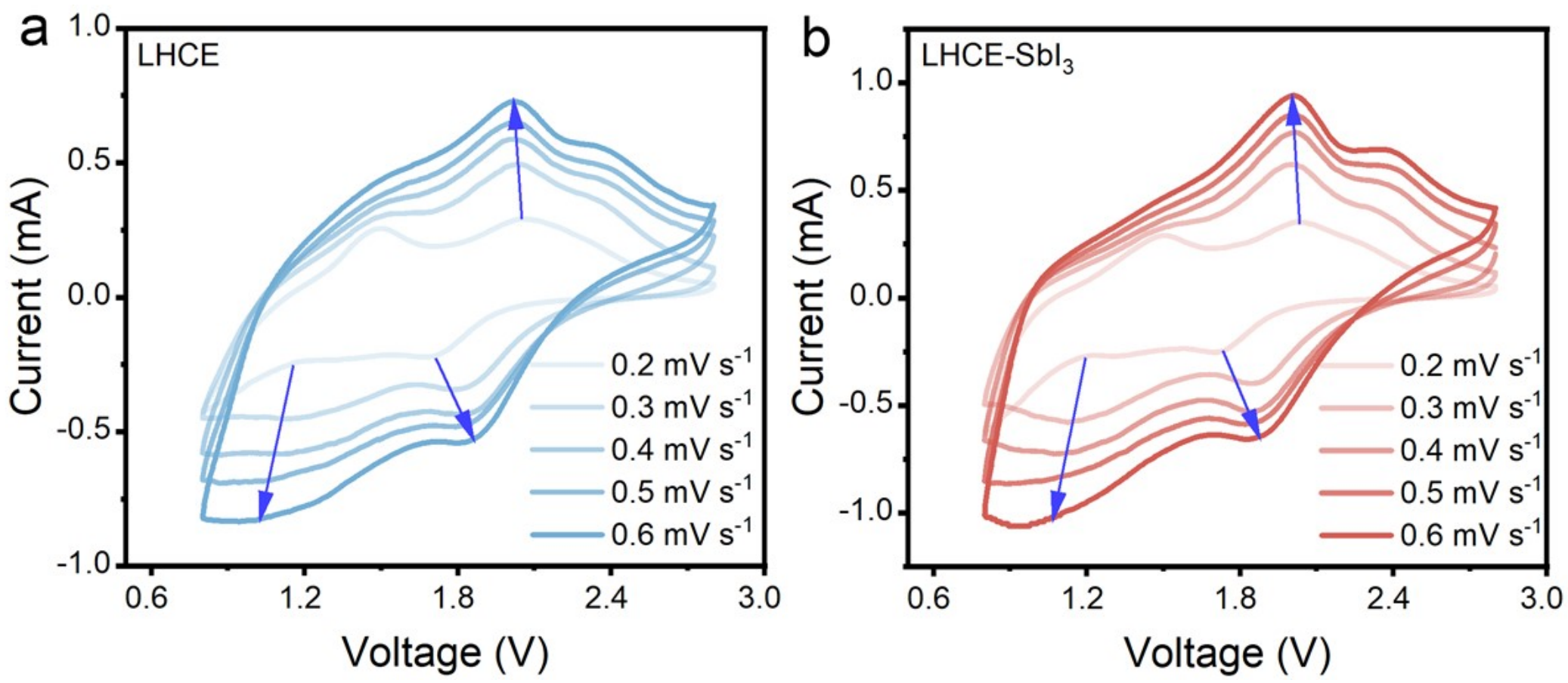


**Figure S11.** CV profiles of the Na-S cells with different LHCEs at various scan rates.

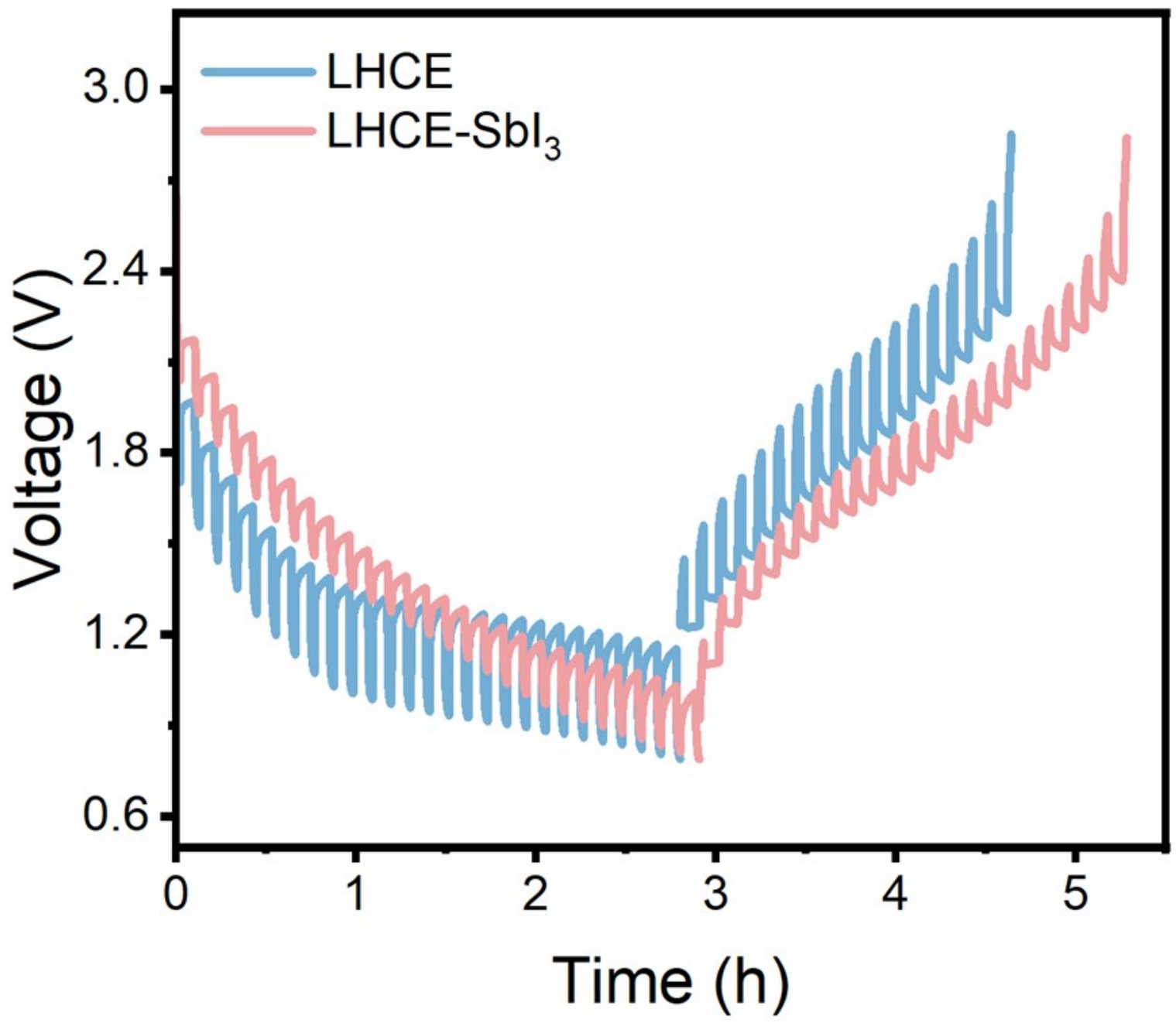


**Figure S12.**GITT voltage profiles of the Na-S cells with different LHCEs as a function of time.

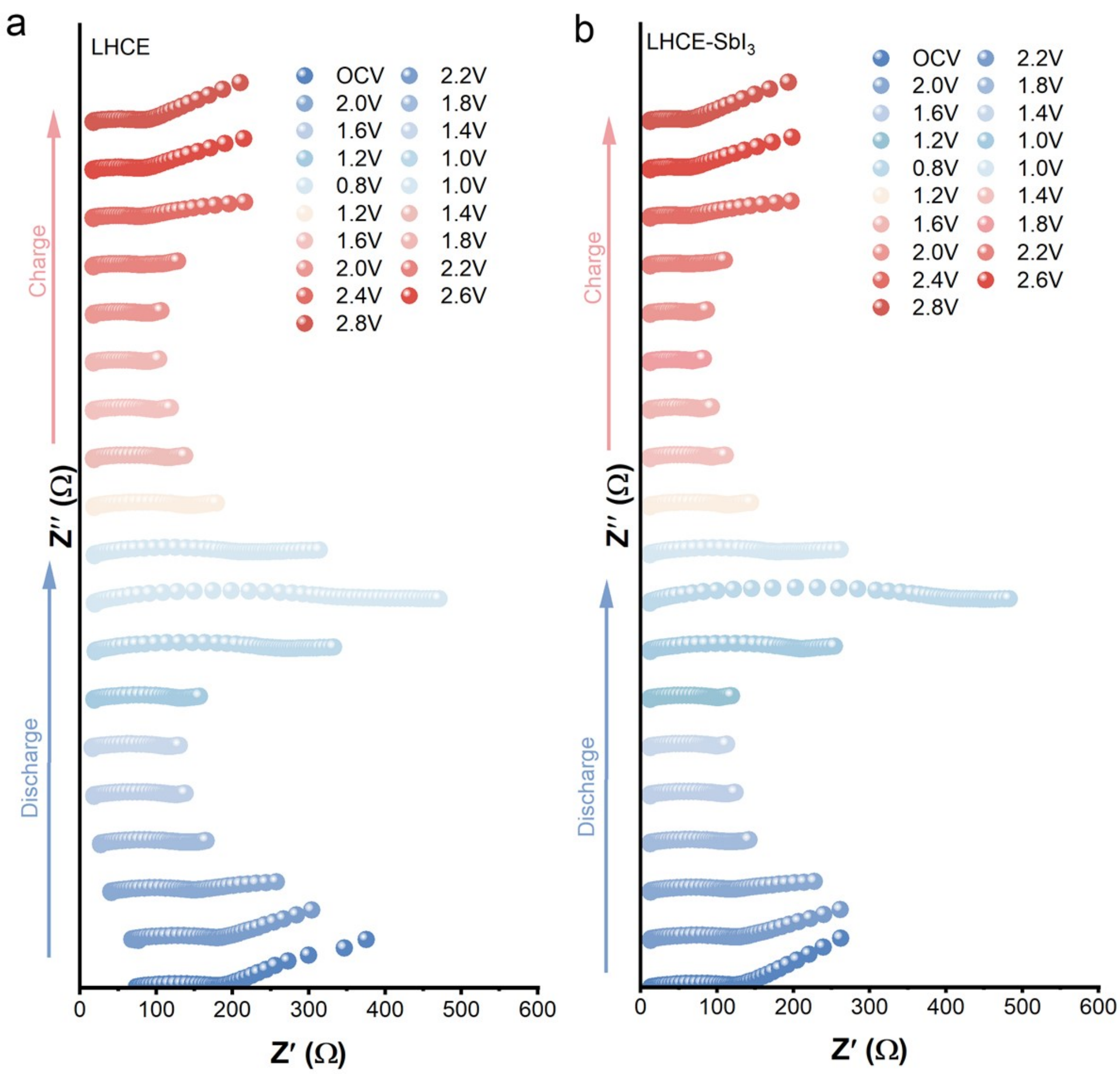


**Figure S13.**In-situ EIS curves for the LHCE and LHCE-$SbI_3$ Na-S cells.

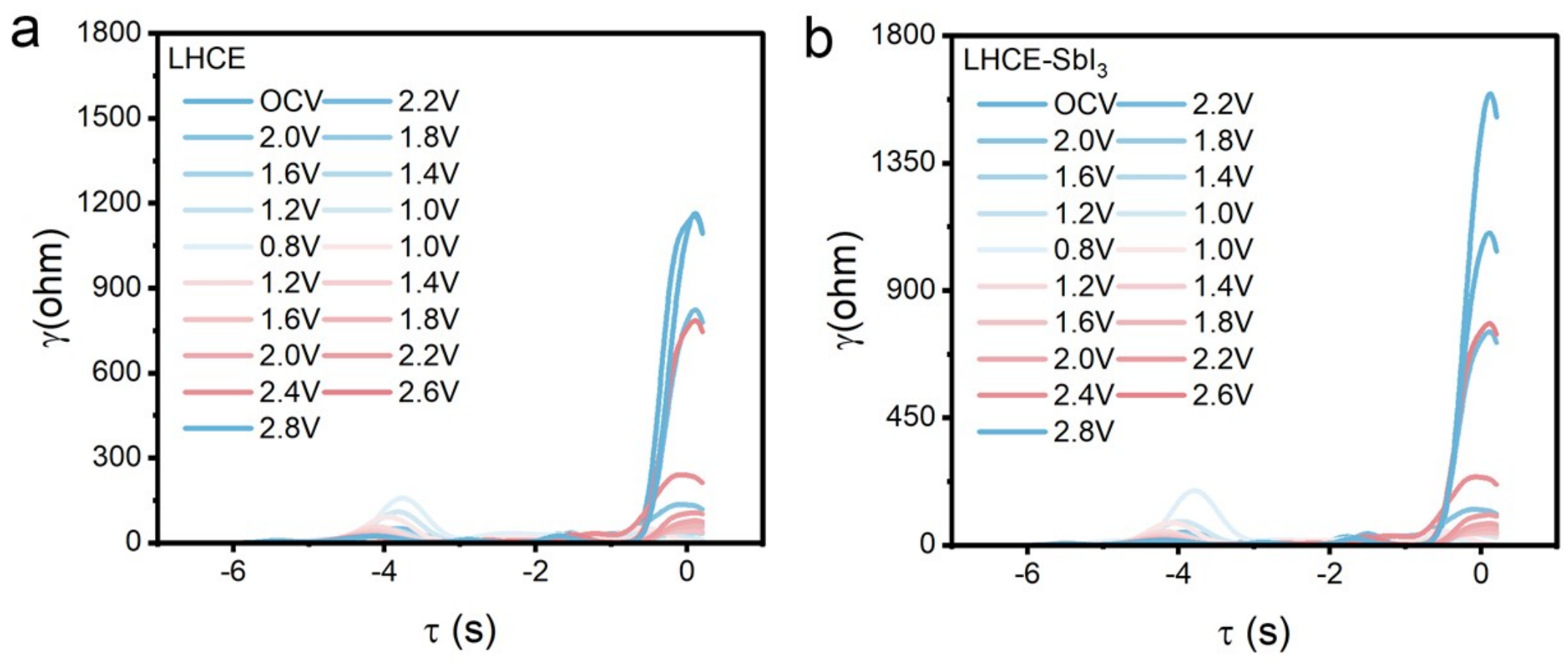


**Figure S14.**Decoupling of DRT peak derived from the in-situ EIS curves.

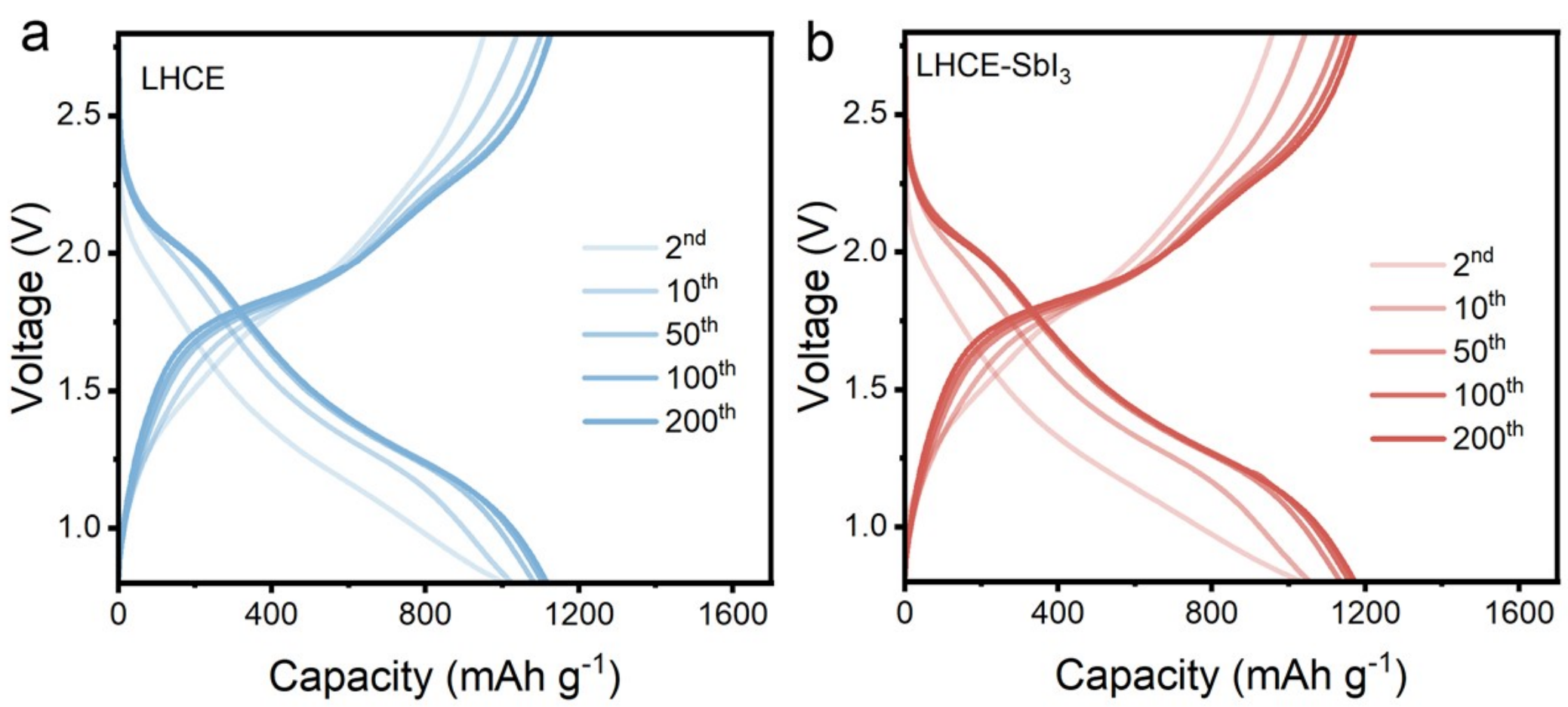


**Figure S15.**The discharge-charge profiles at the selected cycles at 0.2 C.

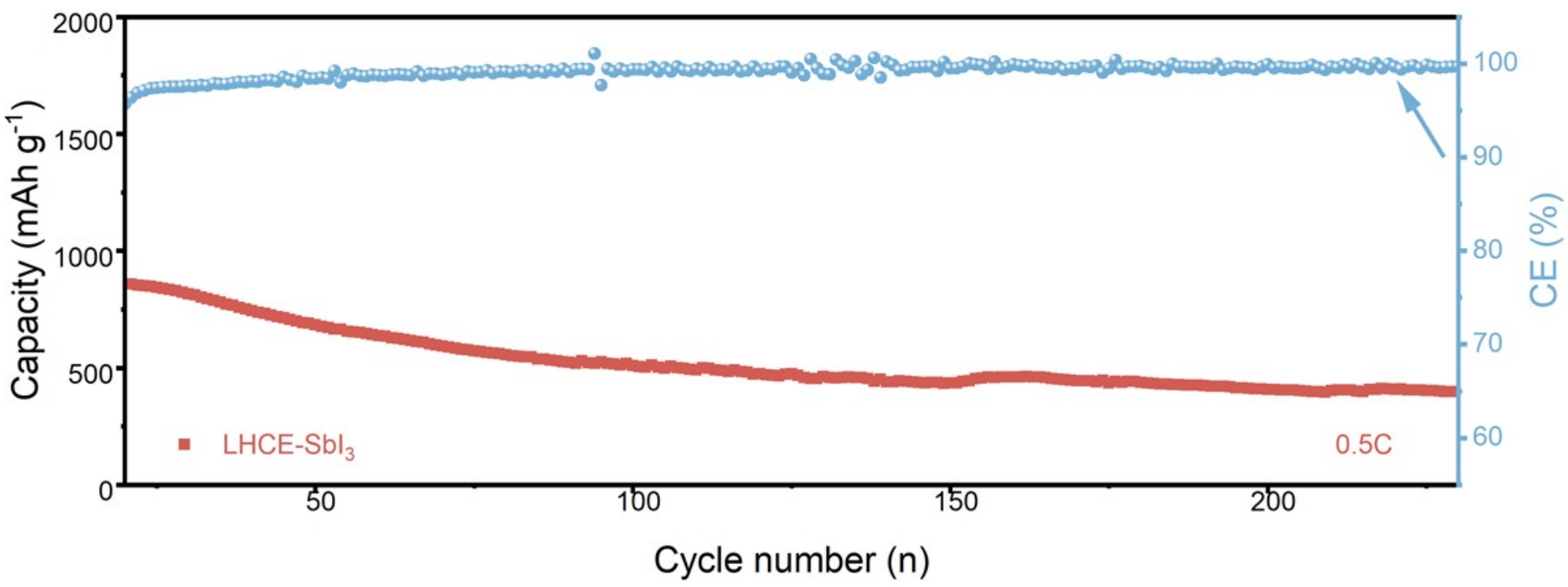


**Figure S16.**Cycling performance of the LHCE-$SbI_3$ cell with a high areal loading of 2mg $cm^{-2}$ at 0.5 C.

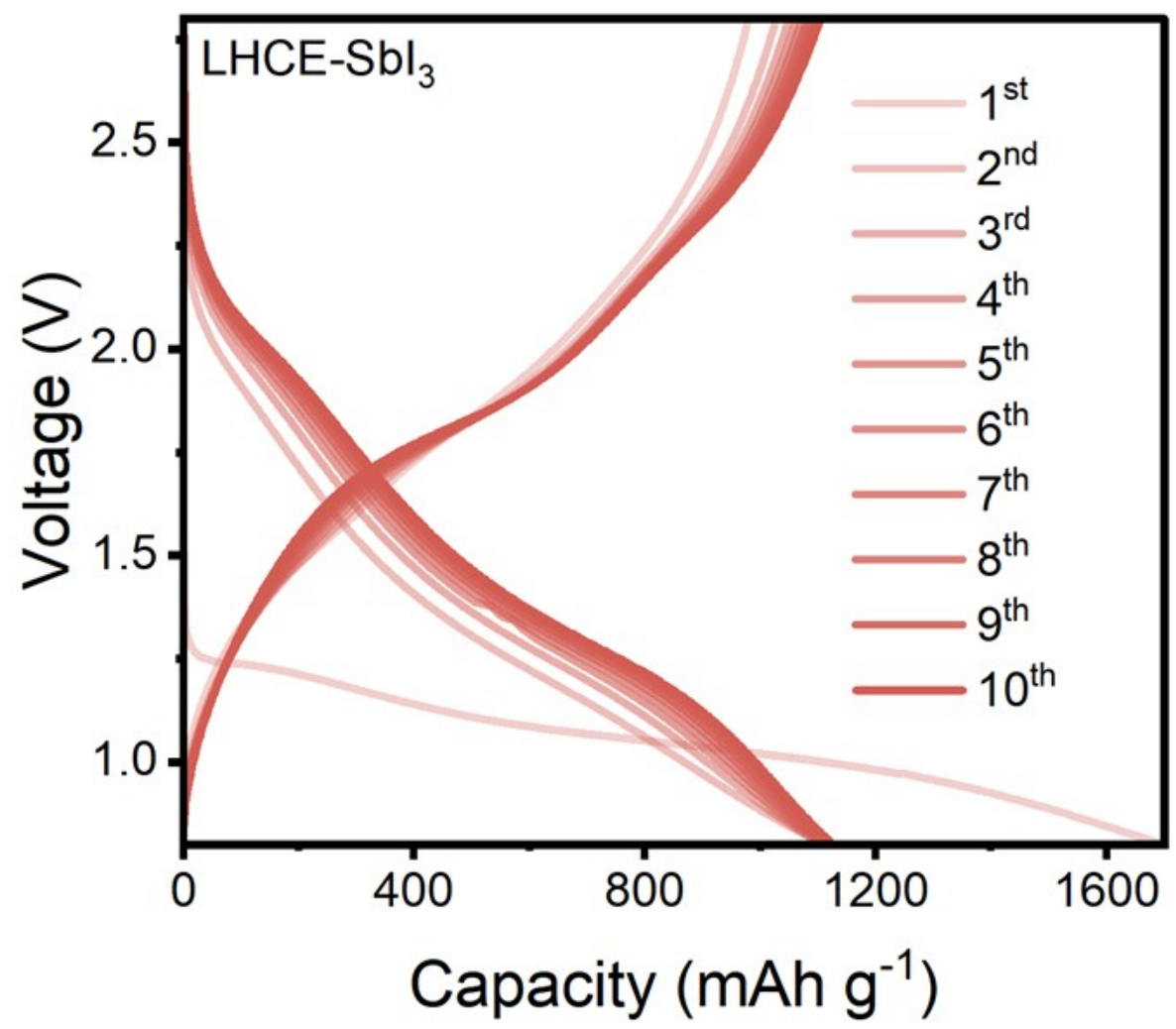


**Figure S17.**Charge-discharge profiles of the initial 10 cycles at 0.2 C prior to long-term cycling at 1.0 C.
